# Elevated mevalonolactone from *Ruminococcus torques* contributes to metabolically unhealthy obesity development

**DOI:** 10.1016/j.jbc.2025.110281

**Published:** 2025-05-22

**Authors:** Hong-Yu Nie, Meng-Fei Zhao, Tian-Yu Wu, Ming-Jie Zou, Yi-Ping Tang, Xiao-Chen Wang, Nan-Nan Wang, Zi-Yue Zhou, Yan Bi, Yue Zhao, Xi-Tai Sun, Jing-Zi Zhang, Lei Fang, Chao-Jun Li

**Affiliations:** 1 Ministry of Education Key Laboratory of Model Animal for Disease Study, Model Animal Research Center of the Medical School, Nanjing University, Nanjing, Jiangsu Province, China; 2 Department of Hepatobiliary Surgery, Affiliated Drum Tower Hospital, Medical School of Nanjing University, Nanjing, China; 3 State Key Laboratory of Reproductive Medicine and Offspring Heath, Nanjing Medical University, Nanjing, China

**Keywords:** metabolically unhealthy obesity, metabolically healthy obesity, geranylgeranyl pyrophosphate (GGPP), mevalonolactone (MVL), Ruminococcus torques (*R.torques*)

## Abstract

Obese individuals are categorized as either “Metabolically Unhealthy Obesity” (MUO) or “Metabolically Healthy Obesity” (MHO) based on their insulin resistance and metabolic disorders. However, the intrinsic mechanism remains largely unknown. By examining gut microbiota and fecal metabolome of patients with MUO and MHO, we identified intestinal microorganism *Ruminococcus torques* (*R. torques*) and its metabolite mevalonolactone (MVL) as risk factors for insulin resistance and metabolic disorders. Both *R. torques* and MVL administration result in the MUO phenotype in mice. In general, MVL is an intermediate metabolite in the eukaryotic mevalonate (MVA) pathway; however, we found that the prokaryote *R. torques* has the potential to produce MVL. We further showed that MVL could directly bind to the transcription factor ZNF384, triggering its nucleation and subsequent binding to the promoter regions of *GGPPS*. GGPPS enhances Ras prenylation and promotes insulin resistance. In conclusion, the abnormal colonization of *R. torques* in the gut leads to an increased level of MVL in patients. This, in turn, affects the expression of *GGPPSvia* ZNF384, ultimately contributing to the development of MUO.

The increasing prevalence of obesity, driven by better living conditions and higher caloric intake, has become a significant global health concern (1). This condition is not uniform, as it presents in various metabolic forms, including metabolically healthy obesity (MHO) and metabolically unhealthy obesity (MUO) (2), with the latter affecting approximately 70% of obese individuals and marked by insulin resistance, abnormal fat storage, and chronic inflammation (3, 4, 5). Understanding these distinct metabolic states is crucial for developing effective prevention strategies.

The gut microbiota plays a pivotal role in metabolic disorders (6, 7). For instance, a shift in the Firmicutes-to-Bacteroidetes ratio, commonly observed in patients with diabetes, is linked to metabolic disruptions and higher blood glucose levels (8, 9). Beneficial bacteria such as *Akkermansia muciniphila* and *Faecalibacterium prausnitzii*, which support gut barrier integrity and anti-inflammatory effects, are often depleted in patients with type 2 diabetes mellitus (T2DM) (10, 11, 12). Additionally, butyrate-producing bacteria like *Roseburia*, *Ruminococcus*, and *Eubacterium rectale*, vital for colon health, are also reduced in these individuals (6, 13). Conversely, bacteria like *Lactobacillus* and *Bifidobacterium*, which can improve intestinal barrier functions and reduce inflammation, may enhance insulin sensitivity (14, 15). *Clostridium* species have been shown to improve circulating glucose levels, reduce systemic insulin resistance and inflammation, increase mitochondrial metabolism, and decrease gut disruption (16).

The health effects of gut bacteria are largely mediated through the metabolites they produce, which are vital biochemical mediators that influence various aspects of human physiology, including immune modulation, energy metabolism, and disease pathology. Short-chain fatty acids (SCFAs), such as butyrate, acetate, and propionate, are key metabolites produced by the fermentation of dietary fibers by gut microbiota and have numerous beneficial effects on the host, including enhancing insulin sensitivity, regulating inflammatory responses, and maintaining gut barrier integrity (17, 18). Indolepropionic acid, produced through bacterial catabolism of tryptophan, has been associated with a reduced risk of developing T2DM, possibly due to its antioxidant properties and protection of pancreatic beta-cells (19). Bile acids, transformed by gut bacteria from primary to secondary forms, affect glucose and lipid metabolism through receptors like FXR and TGR-5, linking gut microbial activities with metabolic health (20, 21).

Building on our previous findings linking the gut microbiota, its metabolites, and insulin resistance (22), we hypothesize that there may be unique gut microbiota characteristics and differences in metabolite levels between MHO and MUO individuals. To identify factors that trigger different metabolic phenotypes in patients with MUO compared to MHO patients, we employed metabolomics and 16S rRNA sequencing to analyze fetal metabolomes and gut microbiomes. This study aims to uncover metabolite and microbial changes in MUO and provide insights for future clinical therapeutic target discovery.

## Results

### Anthropometric and biochemical parameters of the study participants

The study involved 88 patients (average age of 38 ± 22 years) who underwent laparoscopic Roux-en-Y gastric bypass surgery. Table 1 presents the cohort's characteristics, divided according to insulin sensitivity. Among the participants, 36 metabolically unhealthy obesity (MUO) patients exhibited insulin resistance, and 52 metabolically healthy obesity (MHO) did not show insulin resistance. After a 75-g glucose load during the oral glucose tolerance test (OGTT), MUO patients displayed considerably higher levels of fasting blood glucose, 1-h blood glucose, and 2-h blood glucose compared to MHOs without insulin resistance. Likewise, fasting insulin and HOMA-IR were significantly increased in patients with MUO. Notwithstanding similar total body fat content, patients with MUO demonstrated a higher degree of liver fat, visceral fat, and mean adipocyte size. In contrast, subcutaneous fat showed no difference between the two groups. Furthermore, patients with MUO had significantly higher serum ALT and AST compared with MHOs. After excluding the patients treated with lipid-lowering medications, patients with MUO presented higher fasting triglycerides compared to MHOs. There was no observable difference in high-density lipoprotein and low-density lipoprotein levels. Altogether, these findings indicate significant disparities in glucose, insulin, and HOMA-IR levels, as well as in the deposition of triglycerides in insulin target organs, between MUOs and MHOs.

**Table 1:** Anthropometric and biochemical parameters of the study subject^a^

Parameters	MHO (n = 52)	MUO (n = 36)	P
Gender (male) Age (years)	24 42.5 ± 26.5	18 41 ± 18	NS NS
BMI (kg/m^2^)	42.1 ± 11.9	43 ± 16.3	NS
Fasting Glucose (mmol/L)^b^	5.16 (4.06–5.99)	7.28 (4.8–8.73)	<0.0001∗∗∗∗
OGTT 1-h Blood glucose (mmol/L)	8.31 ± 3.89	13.22 ± 2.88	<0.0001∗∗∗∗
OGTT 2-h Blood glucose (mmol/L)	7.3 ± 3	14.8 ± 3.8	<0.0001∗∗∗∗
Fasting Insulin (μIU/ml)^b^	14.96 (8–20.16)	22.11 (12.69–40)	<0.007∗∗
HOMA-IR^b^	3.46 (1.5–5.32)	7.64 (2.71–14.56)	<0.0002∗∗∗
C reactive protein (mg/L)^b^	5.11 (1–8.1)	6.31 (3–9.9)	0.04∗
Triglyeerides (mmol/L)^a^^b^	1.33 (0.75–2)	2.11 (0.95–6.69)	0.003∗∗
Total Cholesterol (mmol/L)^a^	5.02 ± 1.71	5.27 ± 1.85	NS
HDL-C (mmol/L)^a^	1.17 ± 0.64	1.17 ± 0.5	NS
LDL-C (mmol/L)^a^	2.59 ± 1.41	2.79 ± 1.33	NS
Liver fat, %	10 ± 8	16 ± 12	0.01 ∗∗
ALT (U/ml)^b^	20.19 (10.4–29.6)	34.79 (20.9–50.5)	<0.0001∗∗∗∗
AST (U/ml)^b^	16.83 (11.5–20.7)	28.45 (14–50.3)	<0.0001∗∗∗∗
Subcutaneous fat (cm^2^)	492 ± 131	512 ± 118	NS
Visceral fat (cm^2^)	237 ± 49	286 ± 66	0.003∗∗
Mean adipose size (μm)^c^	70 ± 11	77 ± 13	0.01∗∗

a Subjects treated with glucose or lipid-lowering medications were excluded.

b Adipocyte size data were available for 53 participants.

c Data are median (interquartile range).

### Increased *Ruminococcus torques* in MUO contributed to insulin resistance and abnormal lipid metabolism

**Figure 1:**
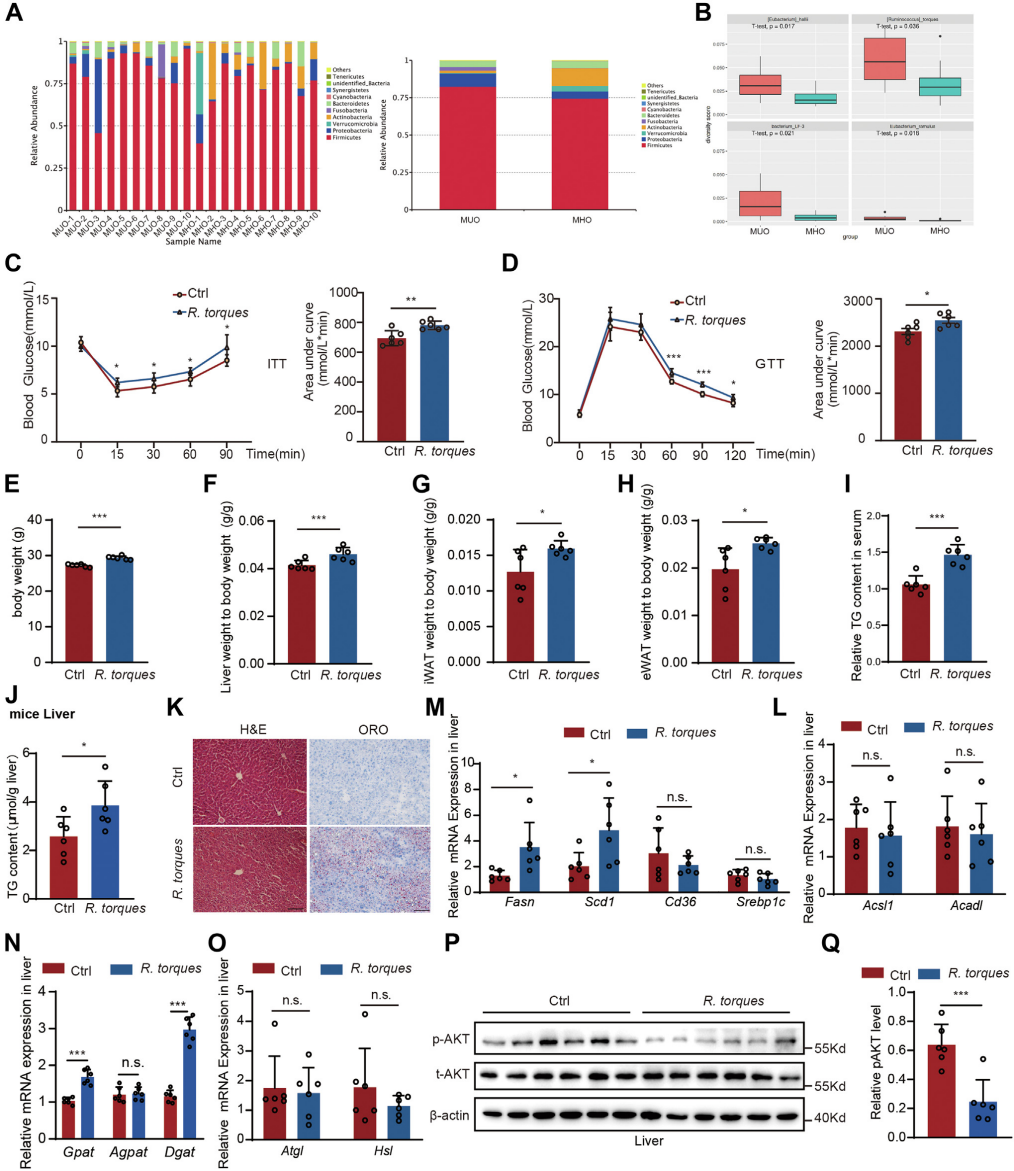
**Increased *Ruminococcus torques* in MUO contributed to insulin resistance and abnormal lipid metabolism**. *A*, differences in MUO and MHO gut microbial species richness at the gate level. *B*, beta diversity indices analyzing MUO and MHO differential microbes. *C–D*, ITT (*C*) and GTT (*D*) in Ctrl and *R. torques*-treated mice (n = 6 mice per group). *E*, body weight of Ctrl and *R. torques*-treated mice with CD-fed (n = 6). *F*, liver weight to body weight ratio in Ctrl and *R. torques*-treated mice (n = 6 mice per group). *G-H*, iWAT and eWAT weight to body weight ratio in Ctrl and *R. torques*-treated mice (n = 6 mice per group). *I*, serum triglyceride (TG) levels in Ctrl and *R. torques*-treated mice (n = 6 mice per group). *J*, liver triglyceride (TG) levels in Ctrl and *R. torques*-treated mice (n = 6 mice per group). *K*, HE staining and oil red staining of liver in mice treated with *R. torques* or not (Scale bar, 50 μm). *M*, expression of genes related to lipogenesis and FFA uptake in the liver of mice treated with Ctrl and *R. torques* (n = 6). *L*, expression of genes related to fatty acid oxidation in the liver of mice treated with PBS and *R. torques* (n = 6). *N-O*, expression of genes related to TG synthesis (N) and lipolysis (*O*) in the liver of mice treated with PBS and *R. torques* (n = 6). *P–Q*, insulin-stimulated phosphorylation of AKT in the hepatocytes treated with *R. torques* (*P*), p-AKT bands were analyzed by densitometry and results were normalized to AKT (*Q*). All experiments were repeated at least twice with similar results. ∗*p* < 0.05, ∗∗*p* < 0.01, ∗∗∗*p* < 0.001; *n.s.*, no significant difference. Data are represented as mean ± SD. Two-sided Student’s *t* test. See also Figures S1 and 2.

To elucidate the alterations in gut microbiota associated with MUO, stool samples from 10 patients per group, as listed in Table 1, were randomly collected and analyzed for microbial diversity using 16S rRNA sequencing. Saturation of observed operational taxonomic units (OTUs) was confirmed in both patients with MUO and MHO by rarity curves and rank abundance plots (Fig. S1*A*), and species accumulation boxplots validated the adequacy of sample sizes (Fig. S1*B*). No significant differences were noted in alpha diversity indexes between the MHO and MUO groups (Fig. S1*C–D*). Comparative analysis of the predominant microbial phyla revealed an increased presence of *Firmicutes, Proteobacteria* and a reduction in *Verrucomicrobia* and *Actinobacteria* in patients with MUO (Figure 1*A*). Further alpha and beta-diversity analyses identified significantly higher levels of specific bacteria such as *R. torques* (*R. torques*), *Eubacterium halli* (*E. halli*), *Eubacterium ramulus*, and *bacterium LF-3* in patients with MUO, suggesting a potential link to MUO pathology (Fig. 1*B*, S1*E*).

*R. torques* was notably increased in patients with MUO, prompting us to investigate its role in MUO development. We administered mice with *R. torques* for 6 weeks and then assessed them against the control groups. Treatment with *R. torques* significantly affected glucose tolerance and insulin tolerance tests (Fig. 1*C* and *D*), without altering fasting or postprandial blood glucose levels (Fig. S1, *F–G*). In the *R. torques*-treated mice, a notable increase in body weight and liver weight was observed (Fig. 1, *E* and *F*), accompanied by significant elevations in both subcutaneous (iWAT) and epididymal (eWAT) fat depots (Fig. 1, *G* and *H*), but there was no significant effect on the weight of the BAT (Fig. S1*J*). Furthermore, the treatment led to elevated triglyceride levels in the blood (Fig. 1*I*) and liver (Fig. 1*J*). There was a marked increase in lipid accumulation in the livers of *R. torques*-treated mice compared to the control mice, as evidenced by the H&E staining and Oil Red O staining (Fig. 1*K*). The treatment notably upregulated the expression of fatty acid synthesis genes (*Fasn, Scd1*) and TG synthesis (*Gpat, Dgat*) in the liver, without significantly impacting fatty acid uptake (*Cd36*), fatty acid oxidation (*Acsl1, Acad*), or lipolysis(*Atgl, Hsl*) (Fig. 1, *M–O*). Moreover, *R. torques* impeded the activation of the hepatic insulin signaling pathway (Fig. 1, *P–Q*) and altered hepatic glucose metabolism, evidenced by reduced pyruvate levels and increased *Hk1* expression (Fig. S1, *H–I*).

In both epididymal white adipose tissue (eWAT) and inguinal white adipose tissue (iWAT), *R. torques* administration elicited distinct molecular and metabolic effects. In eWAT, the treatment upregulated *Scd1* and *Srebp1c* gene expression (Fig. S1*K*), while concurrently downregulating *Hsl* gene expression (Fig. S1*O*). In iWAT, the treatment also upregulated *Fasn*, *Scd1*, and *Srebp1c* gene expression (Fig. S1*L*), indicating enhanced lipogenesis and adipocyte differentiation. However, unlike in eWAT, there were no significant changes in *Hsl* gene expression (Fig. S1*P*) in iWAT. Meanwhile, *Ruminococcus torque* treatment had no significant effect on the fatty acid oxidizing capacity of adipose tissue (Fig. S1*M* and *N*). *R. torques* treatment impaired the insulin signaling pathway in eWAT and iWAT, suggesting a systemic effect on insulin-mediated metabolic pathways (Fig. S1*Q*), and altered eWAT glucose metabolism with incremental pyruvate levels (Fig. S1*R*), but have no change in *Hk1* level (Fig. S1*S*).

To further investigate the functional role of *R. torques*, we administered *R. torques* to high-fat diet (HFD)-fed mice. Under HFD conditions, *R. torques* treatment significantly impaired insulin sensitivity (Fig. S2, *A* and *B*) and reduced glucose homeostasis regulation capacity (Fig. S2, *C* and *D*). The *R. torques*-treated group exhibited significant body weight gain accompanied by increased liver weight and elevated weights of iWAT and eWAT (Fig. S2, *E–H*).

Hepatic analysis revealed that *R. torques* treatment upregulated the expression of fatty acid synthesis-related genes (*Fasn*, *Scd1*, and *Srebp1c*) (Fig. S2*I*) compared to the HFD group, while showing no significant effects on fatty acid uptake markers (*Cd36*, *Fabp5*) (Fig. S2*J*). Concurrently, we observed downregulation of fatty acid oxidation-related genes (*Ppara*, *Cpt1a*) (Fig. S2*K*). Furthermore, *R. torques* administration enhanced triglyceride synthesis-related gene expression (*Gpat*, *Agpat*, *Dgat*) but suppressed lipolysis-associated genes (*Hsl*, *Abhd5*) (Fig. S2, *L* and *M*).

Collectively, our findings demonstrate that *R. torques* treatment induces insulin resistance and disrupts glucolipid metabolic homeostasis in mice under both chow diet (CD) and HFD feeding conditions. These metabolic perturbations manifested through coordinated regulation of lipid anabolic and catabolic pathways, ultimately promoting ectopic lipid accumulation. These findings collectively indicate that *R. torques* treatment causes tissue-specific metabolic dysfunction in the liver and adipose tissue, with distinct responses observed in the liver, eWAT, and iWAT, despite the shared upregulation of key genes involved in lipid metabolism and adipocyte function.

### Elevated levels of mevalonolactone (MVL) were associated with disrupted glucose and lipid metabolism

To identify unique gut metabolites that distinguish MUO from MHO, we employed metabolomics profiling to analyze fecal samples from patients with MUO and MHO. The metabolomic profiling of fetal samples revealed 1457 positive and 954 negative ion peaks, respectively. Unsupervised principal component analysis (PCA) and supervised orthogonal partial least squares discriminant analysis (OPLS-DA) were used to observe general clustering trends and construct classification models, respectively. The distinct separation of MUO and MHO in both PCA and OPLS-DA score plots indicated significant metabolic differences (Fig. S3, *A–D*).

**Figure 2:**
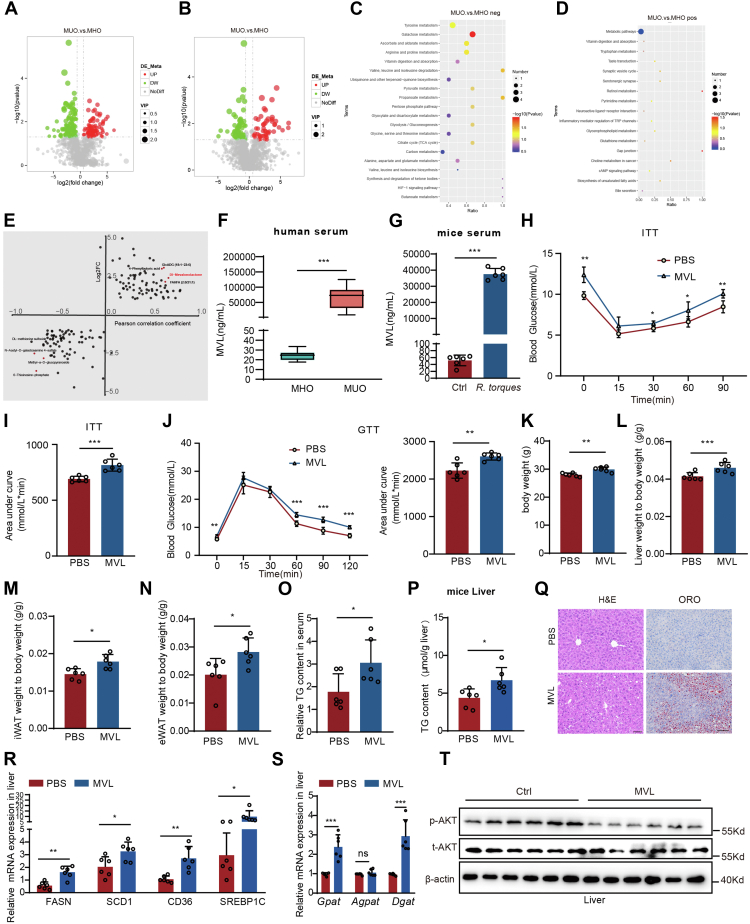
**Elevated levels of mevalonolactone (MVL), induced by *Ruminococcus torques*, were associated with disrupted glucose and lipid metabolism**. *A* and *B*, Volcano plots of the negative iron mode (*A*) and the positive iron mode (*B*) differential metabolites in MUO and MHO. *C*, KEGG with negative metabolome in MUO and MHO patients. *D*, KEGG with positive metabolome in MUO and MHO patients. *E*, the correlation analysis between the content of MVL and the abundance of *R. torques*. *F*, serum MVL levels in MUO and MHO patients (n = 10). *G*, Serum MVL levels in mice treated with *R. torques* (n = 6). *H-J*, ITT (*H* and *I*) and GTT (*J*) in Ctrl and MVL treated mice (n = 6 mice per group). *K–N*, body weight, Liver, iWAT, eWAT weight to body weight ratio in Ctrl and MVL-treated mice (n = 6 mice per group). *O* and *P*, the level of triglyceride (TG) in serum (*O*) and liver (*P*) in Ctrl and MVL-treated mice (n = 6 mice per group). *Q*, H&E staining (*left*) and oil red O (ORO) staining (*right*) of the liver in mice treated with MVL or not (Scale bar, 50 μm). *R* and *S*, expression of genes related to lipogenesis, FFA uptake (*R*) and TG synthesis (*S*) in liver of mice treated with PBS and MVL (n = 6). *T*, insulin-stimulated phosphorylation of AKT in the hepatocytes treated with MVL. p-AKT bands were analyzed by densitometry and results were normalized to AKT (Fig. S3*W*). All experiments were repeated at least twice with similar results. ∗*p* < 0.05, ∗∗*p* < 0.01, ∗∗∗*p* < 0.001; *n.s.*, no significant difference. Data are represented as mean ± SD. Two-sided Student’s *t* test was used. See also Figures S3 and 4.

In positive ion mode, 113 metabolites exhibited differential expression between MUO and MHO, with 52 increased and 61 decreased in MUO. In negative ion mode, 208 metabolites were differentially expressed, with 82 elevated and 126 reduced in MUO compared to MHO. Volcano plots visualized significant differences (Fig. 2, *A* and *B*), while heatmap clustering revealed that MUO shared similar metabolite profiles with MHO in negative ion mode but had unique profiles in positive ion mode (Fig. S3, *E* and *F*). KEGG analysis revealed 17 enriched metabolic pathways in positive mode and 33 in negative mode, with seven pathways showing significant differences, including retinol metabolism, gap junction, galactose metabolism, valine, leucine, and isoleucine degradation, propanoate metabolism, ascorbate and aldarate metabolism, and arginine and proline metabolism (Fig. 2, *C* and *D*).

Previous results have shown that *R. torques* is closely associated with the development of MUO. The content of gut microbiota is closely related to the level of intestinal metabolites. We conducted a correlation analysis between intestinal metabolites and *R. torques* and found that MVL had the highest combined score in terms of change magnitude and correlation with *R. torques* (Fig. 2*E*). Moreover, mass spectrometry confirmed the elevated levels of MVL in the blood samples of patients (Fig. 2*F*). Similarly, we detected abnormally elevated MVL in the blood of *R. torques*-treated mice (Fig. 2*G*), indicating a correlation with *R. torques* abundance.

To investigate the impact of MVL on MUO, mice were treated with MVL for 6 weeks. MVL-treated mice exhibited significantly impaired insulin tolerance and glucose tolerance (Fig. 2*H*–*J*) as well as changes in blood glucose levels (both fasting and postprandial) (Fig. S3, *H* and *I*). Body weight, liver, iWAT, and eWAT weight and triglyceride content were also elevated in the MVL-treated group, along with the presence of lipid drops in the liver (Figs. 2, *K–Q*, and S3*G*). MVL treatment upregulated liver fatty acid synthesis genes (*Fasn, Scd1, Srebp1c*), TG synthesize and increased fatty acid uptake (*Cd36*) (Fig. 2, *R* and *S*), while no significant changes were observed in fatty acid oxidation genes (Fig. S3*J*). Lipolysis-related genes (*Atgl, Hsl*) were significantly decreased (Fig. S3*K*). Additionally, MVL treatment affected hepatic glucose metabolism, with reduced pyruvate (PA) levels and *Hk1* expression (Fig. S3, *L* and *M*). We observed the inhibitory effect of MVL on the activation of the insulin signaling pathway in the liver (Fig. 2*T*), and analyzed at Fig. S3*W*.

In contrast, in iWAT, MVL treatment upregulated lipogenesis genes (*Fasn, Scd1*) (Fig. S3, *N* and *O*). However, no significant effects were observed on lipid oxidation (Fig. S3, *P* and *Q*) in iWAT and eWAT. In eWAT, MVL treatment downregulated expression of lipolysis-related genes, no significant effects were observed in iWAT (Fig. S3, *R* and *S*). *R. torques* treatment altered eWAT glucose metabolism with reduced pyruvate levels (Fig. S3, *T* and *U*) and impaired the insulin signaling pathway in eWAT but not in iWAT (Fig. S3, *V* and *X*).

Under HFD conditions, we observed that MVL-treated mice exhibited reduced insulin sensitivity (Fig. S4*A* and *B*) accompanied by disrupted glucose homeostasis (Fig. S4*C* and *D*). Compared to the HFD group, MVL-treated HFD mice demonstrated more pronounced body weight gain (Fig. S4*E*), along with increased liver weight and elevated masses of iWAT and eWAT (Fig. S4, *F–H*). Hepatic analysis revealed significant upregulation of fatty acid synthesis-related genes (*Fasn, Scd1, and Srebp1c*) (Fig. S4*I*), while fatty acid uptake markers (*Cd36, Fabp5*) remained unchanged (Fig. S4*J*). Notably, fatty acid oxidation-related gene (*Cpt1a*) was significantly downregulated (Fig. S4*K*). Concurrently, triglyceride synthesis-related gene expressions (*Gpat, Agpat, Dgat*) were upregulated alongside suppressed lipolysis-associated genes (*Hsl, Abhd5*) (Fig. S4, *L* and *M*). Consistent with observations in CD-fed mice, MVL treatment similarly exacerbated insulin resistance and aggravated glucolipid metabolic dysregulation in HFD-fed mice.

Overall, these findings suggest that MVL, which is correlated with *R. torques* abundance, plays a role in the development of insulin resistance and metabolic disorders in mice. Elevation of MVL triggered by *R. torques* leads to the occurrence of insulin resistance and disorders in glucose and lipid metabolism.

### MVL alters the metabolic pattern of the liver in mice

**Figure 3:**
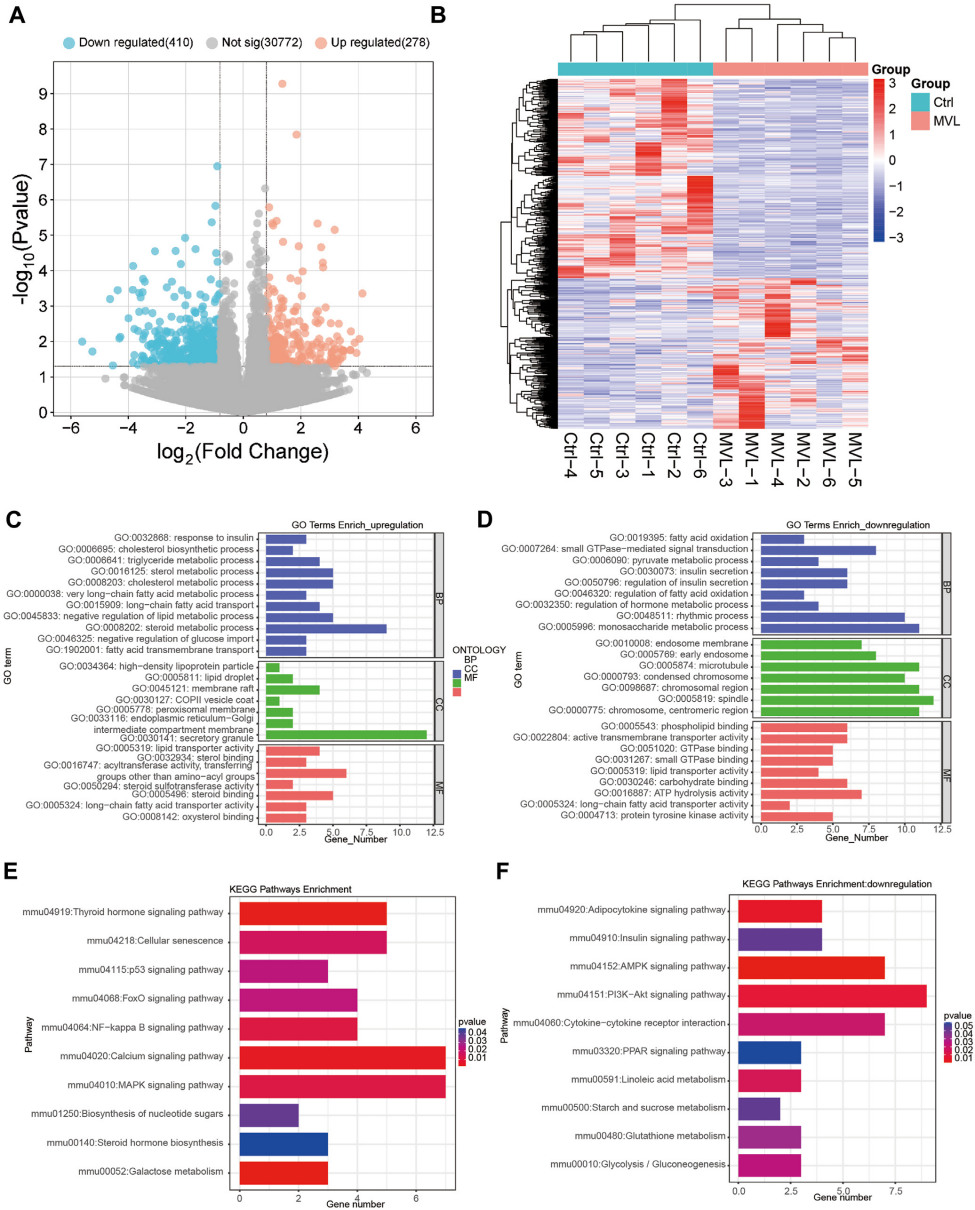
**MVL alters the metabolic pattern of the liver**. *A*, Volcano plot analysis of differential gene expression in the liver transcriptome of mice treated with MVL (n = 6). *B*, Heatmap illustrating the differentially expressed genes in the liver of MVL-treated mice (n = 6). *C* and *D*, functional enrichment analysis of differentially expressed genes in the liver of MVL-treated mice, categorizing biological processes (BP), molecular functions (MF), and cellular components (CC) for genes that are upregulated (*C*) or downregulated (*D*). *E* and *F*, KEGG pathway enrichment analysis of genes that are upregulated (*E*) or downregulated (*F*) in the liver of MVL-treated mice. All experiments were repeated at least twice with similar results. ∗*p* < 0.05, ∗∗*p* < 0.01, ∗∗∗*p* < 0.001; *n.s.*, no significant difference. Data are represented as mean ± SD. Two-sided Student’s *t* test. See also Fig. S5.

To delve deeper into the mechanisms of MVL and metabolic disorders, we administered MVL to mice for a 6-week period and performed transcriptome sequencing on their livers (n = 6). The boxplot of log10 RPKM for the 12 samples is showed in Fig. S5*A*. Successfully aligned reads were further evaluated, indicating that the read coverages of the genomes of most samples were uniform and that there was little 5′or 3′bias in sample (Fig. S5, *A* and *B*). Differential gene expression analysis revealed a substantial transcriptional response to MVL treatment, with 284 genes upregulated and 412 genes downregulated when compared to the control group (Fig. 3, *A* and *B*). A comprehensive analysis of the affected biological processes revealed that MVL treatment specifically targeted genes involved in insulin response (GO:00328768), fatty acid transport (GO:0015909, 001902001), and fatty acid oxidation (GO:0019395, 0046320). Concurrently, MVL treatment increased cholesterol synthesis (GO:0006695) and impacted cholesterol metabolism (GO:00088203) and triglyceride metabolism (GO:00066441). Moreover, MVL treatment influenced sterol metabolism (GO:00116125, 0008202) and pyruvate metabolism (GO:00060090). Notably, MVL treatment also affected signaling pathways mediated by small G proteins (GO:00077264) and altered GTPase binding (GO:00331267, 0051020) in molecular function analyses (Fig. 3, *C* and *D*).

KEGG pathway analysis indicated that MVL treatment affected multiple signaling pathways related to insulin signaling, including the FoxO pathway (mmu04068), the PI3K-AKT pathway (mmu04151), and the AMPK pathway (mmu04152). Furthermore, MVL treatment influenced the NF-κB signaling pathway (mmu040064), the MAPK signaling pathway (mmu040010), and the PPAR signaling pathway (mmu03320), which are not directly insulin-related but can interact with and modulate insulin signaling (Fig. 3, *E* and *F*). These findings collectively suggest that MVL treatment could induce liver insulin resistance and metabolic disturbances through complex transcriptional and signaling pathway alterations.

### MVL promotes *Ggpps* expression, which in turn enhances K-Ras prenylation

**Figure 4:**
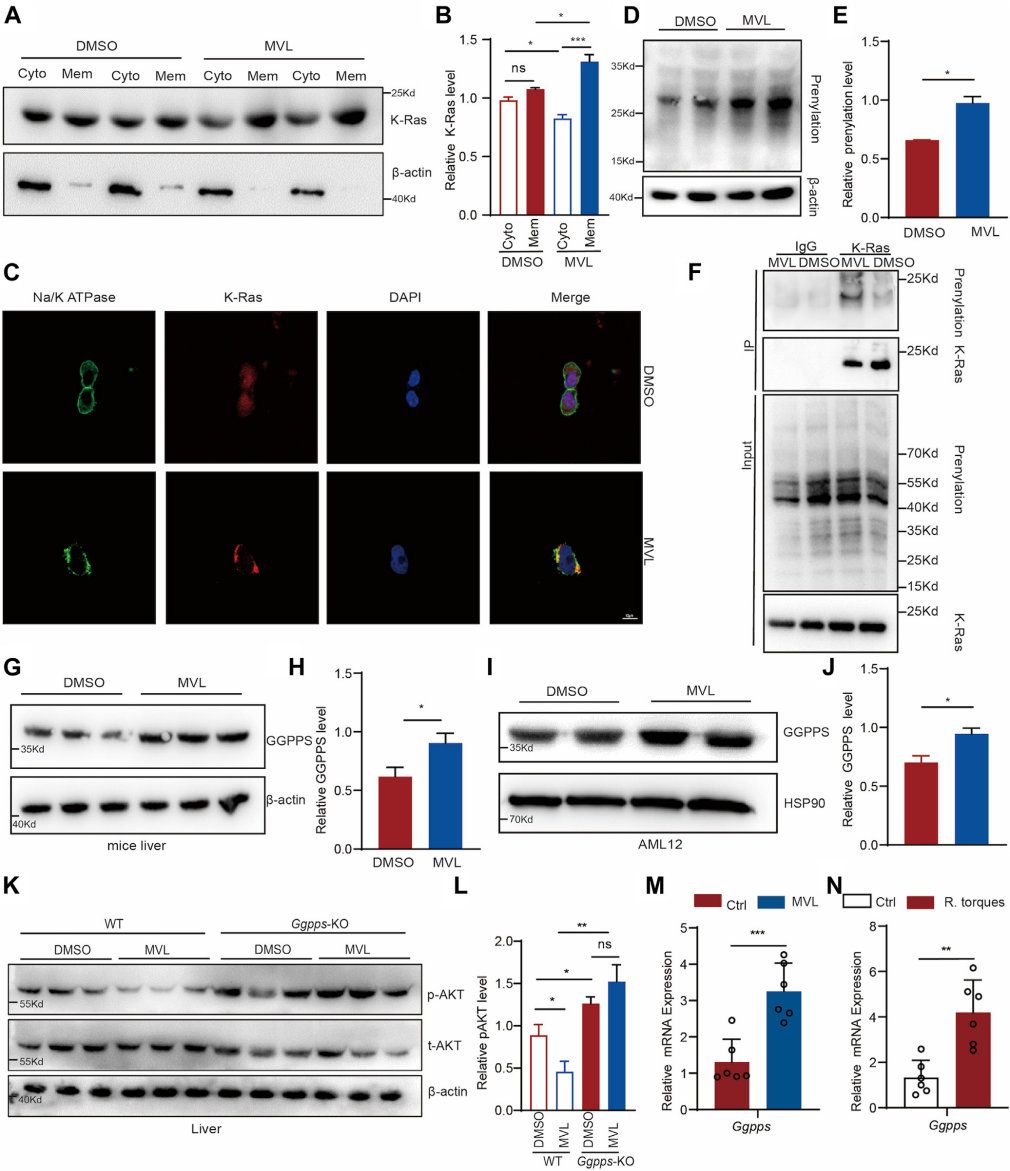
**MVL-induced insulin resistance is dependent on promoting the expression of GGPPS, which in turn enhances K-Ras prenylation**. *A* and *B*, membrane-associated K-Ras (lipid-soluble protein with prenylation in the detergent phase) and cytoplasm-associated K-Ras (water-soluble protein with no prenylation in the aqueous phase) were obtained by Triton X-114 extraction and analyzed by immunoblotting. K-Ras bands were analyzed by densitometry (*B*). *C*, immunofluorescence identifies changes in K-Ras localization before and after MVL treatment (Scale bar, 10 μm). *D* and *E*, the level of protein prenylation in MVL-treated AML12 cells (*D*). Prenylation was analyzed by densitometry and results were normalized to β-actin (*E*). *F*, using anti-K-RAS antibody for immunoprecipitation and detect the level of prenylation. *G* and *H*, the protein level of GGPPS in the liver of MVL-treated mice (*G*). GGPPS bands were analyzed by densitometry and results were normalized to β-actin (*H*). *I* and *J*, the protein level of GGPPS in MVL-treated cells (AML12) (*I*). GGPPS bands were analyzed by densitometry and results were normalized to HSP90 (*J*). *K*, isolate primary liver from *Mx1*-*Ggpps*-KO mice, treat with MVL for 24 h, followed by insulin treatment for 30 min, and then detect the level of p-AKT. p-AKT bands were analyzed by densitometry and results were normalized to AKT (*L*). *M* and *N*, the mRNA level of *Ggpps* in MVL (*G*) and *R. torques* treated mice (*H*) (n = 6). All experiments were repeated at least twice with similar results. ∗*p* < 0.05, ∗∗*p* < 0.01, ∗∗∗*p* < 0.001; *n.s.*, no significant difference. Data are represented as mean ± SD. Two-sided Student’s *t* test was used. See also Fig. S6.

MVL treatment exerted a common influence on multiple signaling pathways, including AMPK, MAPK, PI3K-AKT, and NF-κB as well as affecting small G protein-mediated signaling. These changes led us to investigate K-Ras, a small G protein known to be a common upstream regulator of these pathways. Activation of K-Ras is translocated to membrane depended on its post-prenylation. We observed an increase in the membrane localization of K-Ras following MVL treatment (Fig. 4*A, B*). By immunofluorescence, we also observed that MVL treatment promoted membrane-side aggregation of K-Ras (Fig. 4*C*). As an intermediate in the mevalonate pathway, MVL can enhance the overall level of prenylation (Fig. 4, *D* and *E*). Immunoprecipitation analysis revealed an increase in the prenylation modification of K-RAS after MVL treatment (Fig. 4*F*), indicating that MVL promotes the prenylation of K-Ras.

Prenylation is a post-translational modification that relies on GGPP, which is a product of GGPPS. MVL treatment induced a significant upregulation of Ggpps protein levels in liver (Fig. 4*G*) and AML12 cell line (Fig. 4*H*). It also found the upregulation of GGPPS in white adipose tissue (Fig. S6, *A–C*). Knocking down *Ggpps* in AML12 alleviated the inhibitory effect of MVL on the insulin signaling pathway (Fig. S6*D*). To confirm that MVL's function relies on GGPPS, we have constructed the *Ggpps*-LKO mouse (Fig. S6*E*). Isolation of primary hepatocytes from *Ggpps*-LKO and treated with MVL. We found that MVL could no longer inhibit AKT phosphorylation (Fig. 4, *K* and *L*).

Interestingly, we found that MVL promotes *Ggpps* expression through increasing its mRNA level, a phenomenon observed both after MVL treatment and *R. torques* treatment (Fig. 4, *M* and *N*). Similar effects were observed in cell lines (Fig. S6, *F* and *G*).

These results suggest that MVL-induced insulin resistance is dependent on the increased prenylation modification of K-Ras, which is mediated by the upregulation of *Ggpps* at the transcriptional level. MVL, through its impact on Ggpps expression, influences the downstream signaling pathways that contribute to metabolic dysfunction.

### ZNF384 mediates the regulation of MVL on insulin resistance through controlling the expression of *Ggpps*

**Figure 5:**
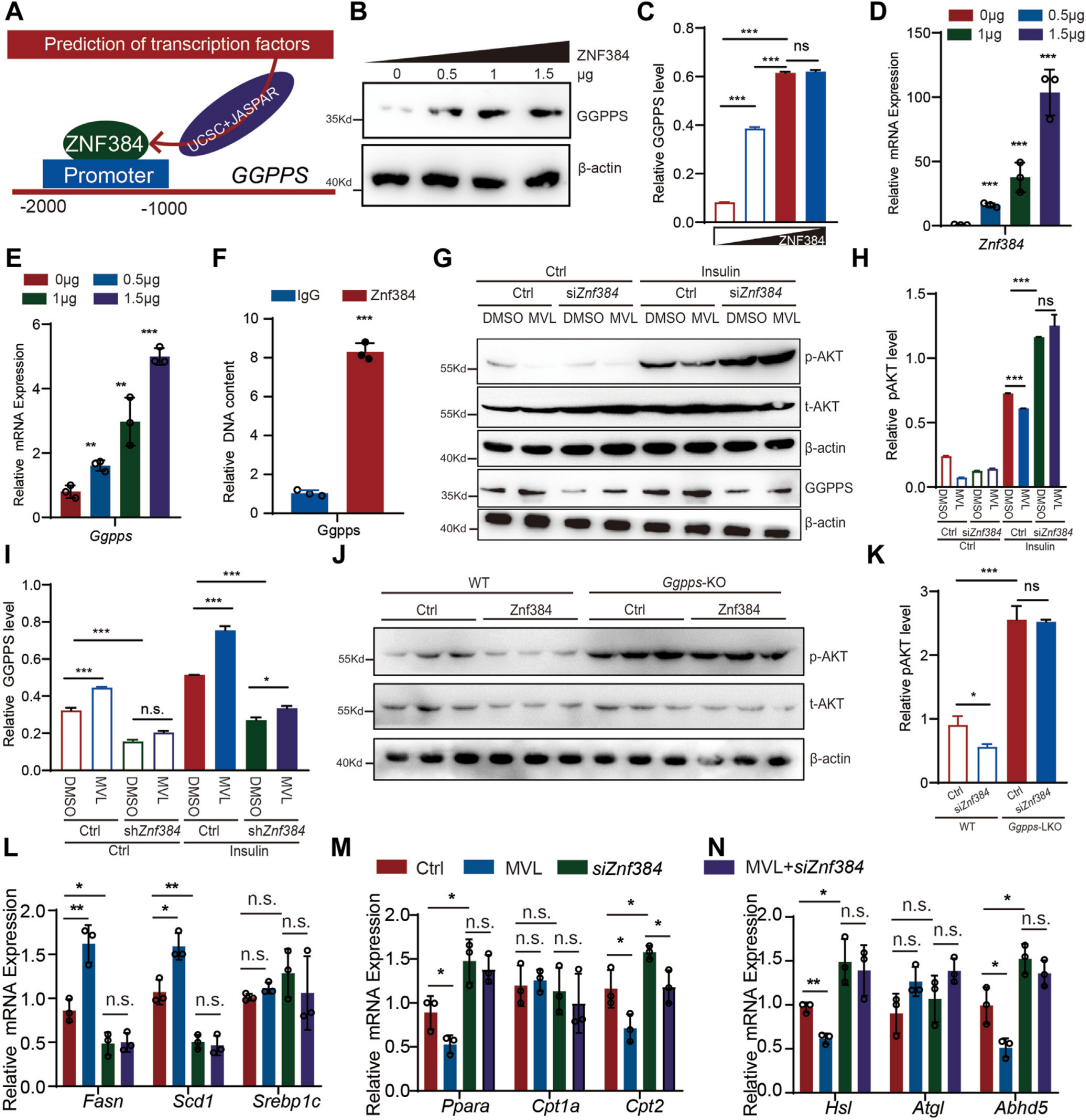
**ZNF384 regulates insulin resistance through controlling the expression of Ggpps**. *A*, prediction of transcription factor binding to the promoter regions of genes *Ggpp*s. *B* and *C*, gradient overexpression of *Znf384* to assess GGPPS protein levels. GGPPS bands were analyzed by densitometry and results were normalized to β-actin (*C*). *D* and *E*, gradient overexpression of *Znf384* to measure mRNA levels of *Ggpps*. *F*, use of ZNF384 antibodies or IgG for ChIP assays to detect the binding of ZNF384 to the promoter regions of predicted target genes. *G–I*, examination of the effects of *Znf384*-KD on the insulin signaling pathway in the presence of MVL (*G*). p-AKT bands were analyzed by densitometry and results were normalized to AKT (*H*). GGPPS bands were normalized to β-actin (*I*). *J–K*, *Ggpps-*KO blocks the inhibition of AKT pathway by ZNF384 in liver (*J*). p-AKT were normalized to AKT (*K*). *L–N*, AML12 cells co-treated with si*Znf384* and MVL were analyzed for *de novo* fatty acid synthesis (*L*), fatty acid oxidation (*M*), and lipolysis (*N*). All experiments were repeated at least twice with similar results. ∗*p* < 0.05, ∗∗*p* < 0.01, ∗∗∗*p* < 0.001, *n.s.*, no significant difference. Data are represented as mean ± SD. Two-way ANOVA or Two-sided Student’s *t* test was used.

In the results of Figure 4, we discovered that MVL influences insulin resistance by affecting the expression of *Ggpps*. This prompted us to hypothesize the existence of a transcription factors modulated by MVL. To identify transcription factors potentially binding to the promoter region of the *Ggpps*, we utilized the JASPAR database, a comprehensive resource for regulatory element databases. By performing a motif search using the promoter sequence of the target gene, predictive analysis identified ZNF384 as a transcription factor binding to the promoter regions of *Ggpps* (Fig. 5*A*, Table 2). Subsequent gradient overexpression of *Znf384* revealed a pronounced dose-dependent increase in the expression of *Ggpps* at both protein and transcription levels (Fig. 5, *B*–*E*). ChIP assays demonstrated that ZNF384 binds strongly to the promoters of *Ggpps* (Fig. 5*F*).

**Table 2:** JASPAR analysis results for ZNF384 binding sites located within the promoter of the *GGPPS* gene

Relative score	Start	End	strand	Sequence
1.00000001	933	944	+	TTTAAAAAAAAA
0.993532855	149	160	+	TTAAAAAAAAAA
0.993532855	934	945	+	TTAAAAAAAAAA
0.993429454	150	161	+	TAAAAAAAAAAA
0.993429454	935	946	+	TAAAAAAAAAAA
0.991393329	936	947	+	AAAAAAAAAAAA
0.983732224	148	159	+	CTTAAAAAAAAA
0.970486628	147	158	+	ACTTAAAAAAAA
0.96656214	725	736	-	TTTAAAAAAATA
0.964999351	932	943	+	GTTTAAAAAAAA
0.957632307	173	184	+	GAAGAAAAAAAA
0.950248214	151	162	+	AAAAAAAAAAAG
0.950248214	937	948	+	AAAAAAAAAAAG

Knocking down *Znf384* (*Znf384*-KD) in cells revealed that MVL impair the insulin signaling in a ZNF384-dependent manner (Fig. 5, *G*–*I*). We overexpress Znf384 in primary hepatocytes from *Ggpps*-LKO and found that Znf384 could not impair the insulin pathway (Fig. 5, *J* and *K*). Moreover, *Znf384*-KD also affected fatty acid synthesis, oxidation and lipolysis (Fig. 5, *L–N*). The findings suggest that ZNF384, as a transcription factor, mediated the regulation of MVL on insulin resistance by controlling the expression of *Ggpps*.

### MVL binding to ZNF384 affects its nuclear localization

**Figure 6:**
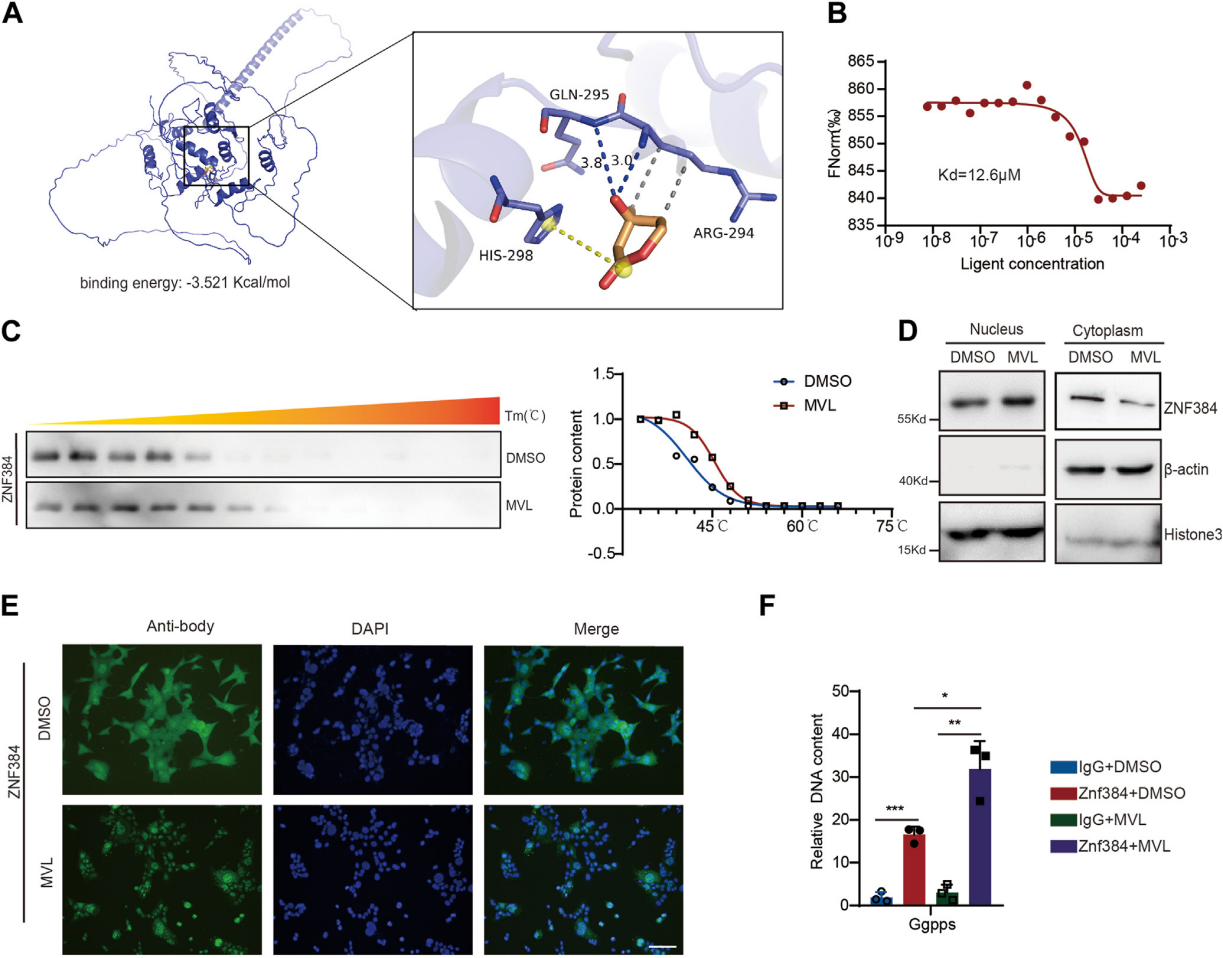
**MVL binding to ZNF384 affects its nuclear localization**. *A*, molecular docking between MVL and Znf384. *B*, microscale thermophoresis (MST) to detect the binding between MVL and Znf384. *C*, analysis of ZNF384 thermal stability changes after MVL treatment using a thermal protein stability assay. *D*, nuclear-cytoplasmic fractionation to study changes in Znf384 localization after MVL treatment. *E*, immunofluorescence to observe Znf384 cellular localization following MVL treatment (Scale bar, 10 μm). *F*, use of ZNF384 antibodies or IgG for ChIP assays to detect the binding of ZNF384 to the promoter regions of *GGPPS*, with and without MVL treatment. All experiments were repeated at least twice with similar results. ∗*p* < 0.05, ∗∗*p* < 0.01, ∗∗∗*p* < 0.001, *n.s.*, no significant difference. Data are represented as mean ± SD. Two-way ANOVA or Two-sided Student’s *t* test was used. See also Fig. S7.

Our findings suggest that MVL alters the expression of a series of downstream target genes by affecting the function of ZNF384, which intrigued us to examine how MVL interacts with ZNF384. Small molecules and proteins primarily interacted through either covalent modification or binding. We employed molecular docking to simulate the binding state of MVL with ZNF384 (Fig. S7*A*). The results indicated that MVL effectively binds to the active site of the protein with a binding energy of −3.5 kcal/mol, suggested that the compound can interact well with the protein’s binding pocket through hydrogen bonding, hydrophobic interactions, and salt bridge interactions. Our analysis of the three-dimensional interactions revealed that MVL forms hydrogen bonds with ZNF384’s GLN295 amino acid, with bond lengths of 3.8 and 3.0. The compound also engages in hydrophobic interactions with the protein’s ARG294 and forms salt bridge interactions with HIS298, thus facilitating the binding of MVL to ZNF384’s active site to form a complex (Fig. 6*A*). Additionally, Microscale Thermophoresis (MST) confirmed the binding of MVL to ZNF384 with a Kd value of 12.6 μM (Fig. 6*B*). Thermal stability analysis further indicated that MVL affects the thermal stability of ZNF384, suggesting MVL binding with ZNF384 (Fig. 6*C*).

To find out the effects to ZNF384 after binding with MVL, we detected the expression of ZNF384 after MVL treatment and found no significant changes (Fig. S7, *B* and *C*). This indicates that MVL's influence on ZNF384 does not involve altering the level of ZNF384. As a transcription factor, ZNF384 functions by entering the nucleus to bind target sequences. Through nuclear-cytoplasmic separation, we observed an increase in nuclear location of ZNF384 upon MVL treatment (Fig. 6*D*). Similarly, through immunofluorescence, we can also observe an increase in nuclear localization of ZNF84 after MVL treatment (Fig. 6*E*). Chromatin Immunoprecipitation (ChIP) assays showed that MVL treatment enhances the binding of ZNF384 to its downstream genes *Ggpps* (Fig. 6*F*). This suggests that the binding of MVL not only enhances the nuclear localization of ZNF384 but also strengthens its interaction with target genes. In summary, our study reveals that MVL promotes the nuclear localization of ZNF384 and enhances its binding to target gene sequences through direct interaction.

## Discussion

MUO (metabolically unhealthy obesity) individuals are characterized by high liver fat content and visceral fat, while MHO (metabolically healthy obesity) individuals exhibit higher insulin sensitivity, better insulin secretion, and more subcutaneous fat content (4, 23). The distinct body fat distributions contribute to the lower susceptibility of MHO individuals to diabetes and atherosclerosis (24). However, it is important to note that the phenotypes of MUO and MHO are not stable. A study showed that 30% of patients with MHO convert to MUO within 6 years (25). Conversely, interventions such as caloric restriction and increased physical activity can lead to the conversion of MUO to MHO (26). These findings suggest that interventions targeting the conversion of MUO to MHO have the potential to improve the metabolic status of patients. Therefore, it is crucial to understand the differences between MUO and MHO to develop effective strategies for intervention.

Existing literature suggests potential links between MUO and factors such as genetics (27), gender disparities (28), and systemic inflammation (29). Although we did not observe significant gender differences in MHO/MUO in the samples collected, probably due to the small sample size. Dietary factors also play a significant role, as consuming medium-chain triglycerides and polyunsaturated fatty acids has been shown to promote a switch from MUO to MHO (30, 31). Amino acid metabolism disturbances are associated with an increased risk of MUO (32). Additionally, impaired adipose tissue proteasome function and enhanced mitochondrial respiratory capacity contribute to the development of MUO (33, 34). Despite these findings, there is still a significant lack of in-depth research in this area. A comprehensive understanding of the molecular mechanisms underlying MUO is crucial for the development of effective pharmaceutical interventions.

Through gut microbiota sequencing, we identified four bacteria, *R. torques*, *E. halli*, *bacterium_LF3*, and *E. ramulus*, showing significantly higher levels in patients with MUO. In contrast, a previous study on the gut microbiota of patients with MUO in the Kumejima of Japan showed significantly higher levels of genera such as g_*Succinivibrio*, g_*Granulicatella*, g_*Brachyspira*, g_*Oribacterium*, and g_*Atopobium* compared to MHO (35). This discrepancy may be attributed to differences in the dietary patterns between the two regions. The Chinese population, in contrast to the Western dietary pattern in Kumejima, consumes a high amount of starch and oil (36). Dietary differences have been shown to have a significant impact on the gut microbiota (37), and variations in the types and proportions of carbohydrates and fats consumed are closely associated with the classification of MUO and MHO (31, 38, 39). Furthermore, a separate study on obese patients in China revealed similar findings to ours, with an overabundance of *R. torques* and Eubacterium halli (40). This further confirms the variations in gut microbiota between different regions and dietary patterns. (37, 40). Therefore, different dietary habits may contribute to the classification of MHO and MUO by influencing the gut microbiota.

Our findings indicate that an overabundance of *R. torques* significantly contributes to dysregulated glucose and lipid metabolism. This dysregulation is linked to insulin resistance. Multi-omics analysis by Jinghua Qin *et al.* also supports a positive association between *R. torques* and elevated TG and TC levels (41). *R. torques*, a gram-positive bacterium known for its mucin degradation capabilities, is considered to be one of the bacterial species that compromises gut barrier integrity. The role of *R. torques* in maintaining metabolic homeostasis appears to be complex. In addition to its involvement in insulin resistance (40, 41, 42), Q. Wu *et al.* found that the effect of *R. torques* on improving insulin resistance depends on the presence of *B. vulgatus* (43). Notably, we did not observe significant changes in *B. vulgatus* in our experiments, which may contribute to the observed differences in results. These changes in the gut microbiota can be influenced by diet, which has a reshaping effect on the gut microbiome (37, 44). In concurrent studies conducted by others, an increase in *R. torques* under fasting conditions was found to be beneficial for the accumulation of liver lipid droplets (45).

In our quest to understand how gut microbiota contributes to obesity, we conducted a metabolomic analysis to uncover the metabolic changes in a patient group exhibiting increased levels of *R. torques*. We were surprised to observe a substantial increase in mevalonolactone (MVL), which are intermediate molecules in the cholesterol synthesis pathway. This upregulation of MVL was closely associated with the abundance of *R. torques*.

Mevalonolactone (MVL), as an essential component of the mevalonate pathway, may influence the synthesis of downstream products such as geranylgeranyl diphosphate (GGPP) and farnesyl diphosphate (FPP). GGPP, a crucial intermediate in the mevalonate pathway, plays a pivotal role in cholesterol, terpene, and terpenoid synthesis, primarily occurring in the liver (46, 47). GGPP functions through protein prenylation-dependent mechanisms, involving peripheral proteins and signal transduction proteins with cysteine residues typically found in the CaaX motif (48, 49, 50, 51). GGPP also activates KRAS/MEK/ERK signaling, which inhibits the insulin signaling pathway PI3K-AKT, leading to insulin resistance (52, 53, 54).

We observed that MVL influences K-Ras geranylgeranylation by promoting the expression of GGPPS, thus inhibiting AKT activation and promoting insulin resistance. Interestingly, during this process, MVL plays two roles. First, as an intermediate in GGPP synthesis, MVL increases the overall flow of the pathway as a substrate. Secondly, MVL promotes the expression of GGPPS, thereby increasing the valve for GGPP synthesis. Through these two mechanisms, MVL effectively enhances GGPP production. This intriguing regulatory process suggests that metabolites can act in a synergistic manner, exerting their functions rapidly and efficiently through different modes of action.

Overall, MVL can increase GGPPS expression, leading to insulin resistance. What perplexes us is the mechanisms by which MVL upregulates Ggpps expression. In our previous study, we identified EGR1 as a transcription factor of *Ggpps*, playing a vital role in the development of insulin resistance (55), which reminds us. To explore this potential mechanism, we predicted the transcription factors for GGPPS and identified a transcription factor, ZNF384. This discovery is intriguing as it suggests that MVL may impact insulin resistance by influencing this particular molecule. ZNF384 was initially identified as CIZ, a protein binding to the SH3 domain of p130Cas in rats (56). It binds to an A-rich DNA consensus sequence and regulates the transcription of matrix metalloproteinases MMP-1, MMP-3, and MMP-7 (56, 57) and type I collagen (58). In our experiment, we found that MVL promotes the nuclear localization of ZNF384 rather than increasing the expression of ZNF384 to enhance GGPPS expression. The binding of MVL may explain the mechanism underlying the promotion of ZNF384 nuclear translocation.

**Figure 7:**
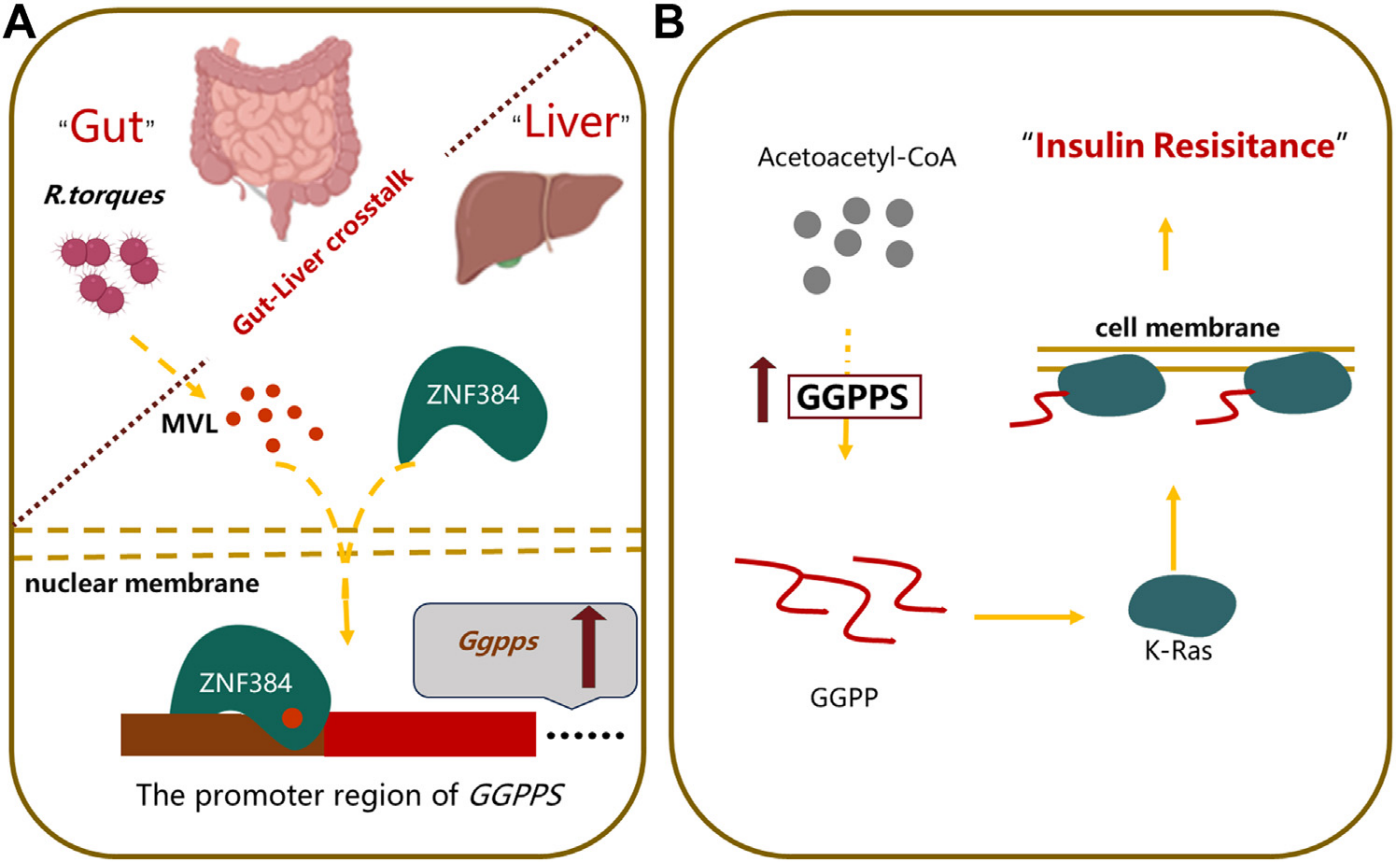
**MVL driven by *R. torques* affects insulin resistance and cholesterol synthesis associated with MUO**. An increase in *R. torques* proliferation caused by a dysbiosis of the gut microbiota, leading to an increase in the body's MVL content. The aberrant upregulation of MVL interacts with ZNF384, facilitating its nuclear translocation (*A*). ZNF384 targets the promoter regions of *Ggpps*, thereby augmenting their expression. The overexpression of GGPPS modulates insulin resistance through the prenylation of K-Ras (*B*).

In summary, in this study, we used metabolomics and 16S rRNA sequencing to explore microbial changes in this patient group and provide insights for subsequent clinical therapeutic targets. We observed high levels of *R. torques* and MVL in MUO patients. We found that the over-colonization of *R. torques* led to increased MVL content in the organism, triggering the increase in nuclear localization of ZNF384 and subsequently increasing the expression of GGPPS, thereby impacting insulin resistance, contributing to the classification of MUO and MHO (Fig. 7).

### Limitations of the study

Despite our findings, our study has certain limitations that should be considered. First, we observed an abnormal increase in *R. torques*, but the reasons for this increase are still unclear. Additionally, although we detected that *R. torques* leads to an increase in MVL, the mechanism of this effect is not clear. In addition to this, while we observed the effects of RT and MVL on insulin resistance and hepatic lipid droplet accumulation in male mice, it is not clear whether we had the same effect on female mice. Finally, we focused on the important events in the occurrence of MUO: insulin resistance. Whether MVL affects other processes in MUO, such as inflammation, is not known.

## Experimental procedures

KEY RESOURCES TABLE

**Table:** 

Reagent or resource	Source	Identifier
Antibodies		
Anti-GGPPS	Santa Cruz Biotechnology	Cat# sc-271680
Anti-Phospho-AKT Ser473	Cell signaling technology	Cat# 4060S
Anti-pan AKT	Cell signaling technology	Cat# 4685
Anti-β-actin	Santa Cruz Biotechnology	Cat# sc-47778
Anti-HSP90	Abclonal	Cat#A5027
Anti-ZNF384	Abclonal	Cat#A15964
Chemicals, Peptides, and Recombinant Proteins		
Paraformaldehyde	Aladdin	Cat #C104190
Insulin used in ITTs	Novo-Nordisk	Cat# NDC 0169–1833–11
TRIzol RNA isolation reagent	ThermoFisher Scientific	Cat# 2183555
iTaq SYBR Green supermix	Bio-Rad	Cat# 172–5125
Exosome-depleted FBS	SBI	Cat# EXO-FBSHI-50A-1
Lipofectamine 2000	ThermoFisher Scientific	Cat# 11668–019
Lipofectamine RNAiMAX reagent	ThermoFisher Scientific	Cat# 13778–075
Starch with Palmitate or Oleat	ReadyDietech	Cat# D12492
TaqMan microRNA reverse transcription kit	ThermoFisher Scientific	Cat#4366597
TaqMan universal master mix II	ThermoFisher Scientific	Cat#4440040
Critical Commercial Assays		
Total Cholesterol Assay Kit	Applygen	Cat# 1016
Deposited Data		
Metabonomics data	MetaboLights	MTBLS7597
Experimental Models: Cell Lines		
3T3-L1 cell line	ATCC Cat#CL173	
H9C2 cell line 293T cell line AML12	ATCC Cat# CBP60588 ATCC Cat#CRL-3216 ATCC Cat#CRL-2254	
Experimental models: Organisms/strains		
Mouse: C57BL/6 (Wt) mice	GemPharmatech	Stock No N000013
Oligonucleotides		
Scramble-sense	GeneRay.Biotech	UUCUCCGAACGUCACGUdTdT
Scramble-antisense	GeneRay.Biotech	ACGUGACACGUUCGGAGAAdTdT
si*Ggpps920*-sense	GeneRay.Biotech	CGCCAGAGAACAGAGAAUATT
si*Ggpps920*-antisense *siZnf384*-sense *siZnf384*-antisense	GeneRay.Biotech GeneRay.Biotech GeneRay.Biotech	UAUUCUCUGUUCUCUGGCGTT CCCAUGAUUGUCUCAGCUCUU AAGAGCUGAGACAAUCAUGGG
*Fas*-F	GeneRay.Biotech	AGGTGGTGATAGCCGGTATGT
Fas-R	GeneRay.Biotech	TGGGTAATCCATAGAGCCCAG
*Scd1*-F	GeneRay.Biotech	TTCTTGCGATACACTCTGGTGC
*Scd1*-R	GeneRay.Biotech	CGGGATTGAATGTTCTTGTCGT
*Acc1*-F	GeneRay.Biotech	GATGAACCATCTCCGTTGGC
*Acc1*-R	GeneRay.Biotech	CCCAATTATGAATCGGGAGTGC
*Atgl*-F	GeneRay.Biotech	TGGATGGCGGCATTTCAGACA
*Atgl*-R	GeneRay.Biotech	TGACGCGAAGCTCGTGGATGTT
*Hsl*-F	GeneRay.Biotech	AAACGCAACGAGACAGGCCTCA
*Hsl*-R	GeneRay.Biotech	ATGCCATGTTGGCCAGAGACGA
*Cd36*-F	GeneRay.Biotech	GAGCAACTGGTGGATGGTTT
*Cd36*-R	GeneRay.Biotech	GCAGAATCAAGGGAGAGCAC
*Fatp2*-F	GeneRay.Biotech	TTCCTGAGGATACAAGATACCATTG
*Fatp2*-R	GeneRay.Biotech	CATGAGCTAAACCACCAGGG
*Fatp5*-F	GeneRay.Biotech	GCTATACCAGCATGTCCGCTC
*Fatp5*-R	GeneRay.Biotech	GTGGTCAGAGATTCCAGGTTCC
*Ppara*-F	GeneRay.Biotech	AACATCGAGTGTCGAATATGTGG
*Ppara*-R	GeneRay.Biotech	AGCCGAATAGTTCGCCGAAAG
*Cpt1a*-F	GeneRay.Biotech	TGAGTGGCGTCCTCTTTGG
*Cpt1a*-R	GeneRay.Biotech	CAGCGAGTAGCGCATAGTG
*Cpt1b*-F	GeneRay.Biotech	CCAAACGTCACTGCCTAAGCT
*Cpt1b*-R	GeneRay.Biotech	GGCCGCACAGAATCCAAGTA
*Cpt2*-F	GeneRay.Biotech	CAACTCGTATACCCAAACCCAGTC
*Cpt2*-R	GeneRay.Biotech	GTTCCCATCTTGATCGAGGACATC
*Acat2*-F	GeneRay.Biotech	CCCGTGGTCATCGTCTCAG
*Acat2*-R	GeneRay.Biotech	GGACAGGGCACCATTGAAGG
*Hmgcs1*-F	GeneRay.Biotech	AACTGGTGCAGAAATCTCTAGC
*Hmgcs1*-R	GeneRay.Biotech	GGTTGAATAGCTCAGAACTAGCC
*Hmgcs2*-F	GeneRay.Biotech	GAAGAGAGCGATGCAGGAAAC
*Hmgcs2*-R	GeneRay.Biotech	GTCCACATATTGGGCTGGAAA
*Mvk*-F	GeneRay.Biotech	GGTGTGGTCGGAACTTCCC
*Mvk*-R	GeneRay.Biotech	CCTTGAGCGGGTTGGAGAC
*Pmvk*-F	GeneRay.Biotech	AAAATCCGGGAAGGACTTCGT
*Pmvk*-R	GeneRay.Biotech	AGAGCACAGATGTTACCTCCA
*Mvd*-F	GeneRay.Biotech	ATGGCCTCAGAAAAGCCTCAG
*Mvd*-R	GeneRay.Biotech	TGGTCGTTTTTAGCTGGTCCT
Software and Algorithms		
ImageJ	NIH	https://imagej.nih.gov/ij/
GraphPad Prism 8 software	Graphpad	https://www.graphpad.com/scientific-software/prism/

### Mice

C57BL/6J mice, acquired from Nanjing University's Model Animal Research Center, were housed in conditions adhering to strict Specific Pathogen Free (SPF) standards, which included a controlled temperature between 20 °C and 22 °C, humidity levels maintained between 50% and 60%, and a consistent 12-h light-dark cycle. They had unrestricted access to a standard lab diet provided by Xietong Bio. Our research strictly followed all applicable guidelines for the ethical treatment of animals and received the necessary authorization from the Animal Care and Use Committee of Nanjing University's Model Animal Research Center (AP number: #CS20).

To investigate the impact of the gut microbiome, 8-week-old male mice were administered a cocktail of antibiotics (Abx: 1 g/L vancomycin, 2 g/L metronidazole, 1 g/L neomycin, 1 g/L ampicillin) to alter their microbial composition. In order to transplant *R. torques*, the mice were pretreated with the antibiotics for 1 week. Subsequently, *R. torques* (BNCC341166) (10^5^ CFU/g mouse) was administered *via* gavage every 2 days for a period of 6 weeks. To examine the effects of MVL, MVL was prepared at a concentration of 10 mmol/L in PBS. 8-weeks-old Male mice were intraperitoneally injected with MVL (10 μl/g of body weight) every 2 days for a period of 6 weeks. As for the HFD group, we utilized 8-weeks-old male mice HFD-fed for 8 weeks before performing the manipulation as described above and kept them HFD chow-fed during the process.

### Human samples

Obese individuals receiving care in the Endocrinology Department at Drum Tower Hospital, affiliated with Nanjing University Medical School, were categorized into Metabolically Healthy Obesity (MHO) and Metabolically Unhealthy Obesity (MUO) groups based on criteria from a prior study. Exclusion criteria ensured participants had not used corticosteroids, glucose, or lipid-lowering medications in the past 3 months, and they were free from acute infections, autoimmune diseases, and significant cardiovascular or cerebrovascular conditions. All participants underwent an Oral Glucose Tolerance Test (OGTT) and provided blood samples before undergoing Roux-en-Y surgery. The Ethical Committee of Drum Tower Hospital, in accordance with the 1975 Declaration of Helsinki, approved the study protocol, ensuring all participants provided written informed consent. The study, bearing the clinical trial number NCT03296605, was registered as an observational study on the International Clinical Trial Registry Platform (ICTRP). Summary of the clinical data for all participants is presented in Table 1.

### Cell culture and differentiation

3T3-L1 preadipocytes (gift from Dr Qi-Qun Tang, Fudan University) were cultured in Dulbecco's modified Eagle's medium (DMEM) with 10% calf serum until 2 days post-confluence. On day 0, cells were induced with DMEM containing 10% fetal bovine serum (FBS), 1 μg/ml insulin, 1 μM dexamethasone, and 0.5 mM three-isobutyl-1-methylxanthine for 2 days. On day 2, cells were then cultured in DMEM containing 10% FBS and 1 μg/ml insulin for another 2 days. Then, the medium was replaced with DMEM containing 10% FBS every other day. Until day 7 or day 8, obvious lipid droplets were observed in the cells, and 3T3-L1 preadipocytes were differentiated into mature adipocytes.

AML12 murine hepatocytes were maintained in DMEM/F12 medium (Gibco) supplemented with 10% fetal bovine serum, 1% penicillin/streptomycin, under standard conditions (37 °C, 5% CO_2_), with subculturing at 80% confluence using 0.25% trypsin-EDTA. Cells were treated with 1 mM MVL for 24 h prior to subsequent experiments.

### Isolation of primary hepatocytes

Primary hepatocytes were isolated using a procedure involving collagenase digestion, initiated through catheterization of the inferior vena cava (IVC) as documented by Li *et al.* (59), 2016. This process began with liver perfusion *via* the IVC for 3 to 4 min following the severance of the portal vein. The liver's color transition to light brown signaled the continuation of perfusion with collagenase for an additional 2 min, closely monitoring for surface cracking indicating digestion completion. Subsequently, perfusion was halted, and the liver was promptly placed into an ice-cold buffer. The liver cells were then strained using a 100 μm nylon mesh and centrifuged at 60 × *g* for 6 min at 4 °C. Following a buffer rinse to remove residual collagenase, cells were resuspended, centrifuged again under the same conditions, and then treated with Percoll before a final centrifugation at 100 × *g* for 10 min. The resulting hepatocyte pellet was once more cleansed in collagenase-free buffer, then cultivated in a medium fortified with penicillin, streptomycin, and 10% fetal bovine serum (FBS). After an overnight incubation, the culture medium was replaced with an exosome-free Dulbecco’s Modified Eagle Medium (DMEM) containing antibiotics, preparing the hepatocytes for further experimental procedures.

### Fetal metabolites extraction and UHPLC-MS/MS analysis

For metabolite analysis, 100 μl of each sample was transferred to EP tubes and vortexed with pre-cooled 80% methanol. Following a 5-min ice incubation, samples were centrifuged at 15,000 g and 4 °C for 20 min. A portion of the resultant supernatant was then diluted to achieve a 53% methanol concentration using LC-MS grade water, transferred to new Eppendorf tubes, and centrifuged again under the same conditions. The clear supernatant obtained was ready for analysis *via* LC-MS/MS.

The UHPLC-MS/MS analysis employed a ThermoFisher Vanquish UHPLC system paired with an Orbitrap Q Exactive HF mass spectrometer, operated by Novogene Co., Ltd in Beijing. The analysis protocol involved loading the samples onto a Hypersil Gold column and executing a 17-min gradient elution at a 0.2 ml/min flow rate. The elution used two solvent systems: A (0.1% formic acid in water) for positive mode and A (5 mM ammonium acetate, pH 9.0) for negative mode, with methanol as solvent B in both instances. The gradient protocol was designed as follows: starting with 2% B, increasing to 100% B over 3 min, maintaining for 7 min, then returning to 2% B. The mass spectrometer settings included a spray voltage of 3.5 kV, capillary temperature of 320 °C, sheath and auxiliary gas flows of 35 psi and 10 L/min respectively, with the S-lens RF level set at 60 and the auxiliary gas heater at 350 °C.

### DNA isolation, library preparation, and 16S rRNA sequencing

Bacterial DNA from a total of 72 samples was extracted utilizing the QIAamp PowerFecal Pro DNA Kit by Qiagen (Qiagen, Hilden, Germany), adhering strictly to the supplied protocols. The integrity and concentration of the isolated DNA were assessed with the Qubit 2.0 fluorometer by Thermo Fisher Scientific (Thermo Fisher ScientificA). To amplify the V3–V4 region of the bacterial 16S rRNA gene, PCR amplification was conducted using primers 341F (5′-CCTACGGGNGGCWGCAG-3′) and 805R (5′-GACTACHVGGGTATCTAATCC-3′). The resulting amplicons were then purified and attached to Nextera XT (Illumina) indexed adapters for sequencing. The KAPA HiFi HotStart ReadyMix (Roche) facilitated all PCR processes. Subsequent quality validation of the purified libraries was performed using the Qubit 2.0 fluorometer and the Agilent 2100 Bioanalyzer system. Sequencing of these libraries produced 250 bp paired-end reads through the Illumina MiSeq platform, employing the MiSeq Reagent Kit v3 for 600 cycles (Illumina, CA, USA). This approach ensured the minimization of batch effects by sequencing all samples concurrently in a single MiSeq run.

### Processing of raw sequencing data

Data analysis and processing were conducted on the Google Cloud platform, starting with the refinement of raw sequencing data. This initial step involved primer removal and filtering out low-quality sequences below a Q30 quality score using the cut adapt tool. Subsequent steps included denoising, pairing of reads, eliminating chimeric sequences, and identifying unique amplicon sequence variants (ASVs) with the DADA2 algorithm implemented in the qiime2 framework. Taxonomic classification of these ASVs utilized the Silva reference database, version silva-138 to 99, incorporating both sequence data and taxonomic information. To distinguish among unclassified ASVs within identical taxonomic groups, a sequential numbering system was applied. The naive Bayes classifier, trained on sequences selected by V3–V4 primers, facilitated the comprehensive taxonomic prediction for each ASV, employing qiime2's feature-classifier plugins. Moreover, the study assessed the microbial communities' functional capabilities through the Phylogenetic Investigation of Communities by Reconstruction of Unobserved States (PICRUSt2), focusing on predicted metabolic pathways from the KEGG database. This comprehensive analysis provided insights into microbial functional diversity and its potential implications.

### MVL level determination

In the preparation phase, liver tissue samples weighing 20 mg each were first homogenized in a solution composed of isopropanol and 100 mM ammonium bicarbonate, mixed at a 1:1 ratio, followed by the addition of 800 μl of methanol. After centrifugation at 18,000 g for 20 min at 4 °C, the supernatants were transferred to new tubes and evaporated under a nitrogen stream. For analysis, the dry samples were reconstituted in 50 μl of 50% methanol and subjected to a final centrifugation before undergoing UPLC-MS/MS analysis.

Quantification of MVLwas performed using an ultra-performance liquid chromatography system paired with tandem mass spectrometry (UPLC-MS/MS, Waters Corp., ACQUITY UPLC H-Class/Xevo G2 TQ-XS). Chromatographic separation was achieved using an ACQUITY BEH C18 column, with a mobile phase of 0.3% ammonia in 10 mM ammonium acetate and 80% acetonitrile containing 50 mM ammonium formate. The operational parameters included a column temperature of 40 °C, sample injection volume of 5 μl, and a flow rate of 0.5 ml/min through a defined gradient. Mass spectrometric detection was conducted in negative ion mode with specified settings for capillary voltage and gas temperatures.

Data analysis involved the use of Masslynx and QuanMET software for peak integration, standard curve calibration, and metabolite quantification, establishing a linear correlation between instrument response and analyte concentration for precise determination of MVL levels (60).

### Glucose tolerance and insulin tolerance tests

To assess glucose metabolism, mice were subjected to glucose tolerance tests where, following a 12-h fasting period, a single intraperitoneal injection of dextrose (1 g/kg body weight) was administered. Blood glucose levels were subsequently determined at intervals of 0 (pre-injection), 30, 60, 90, and 120 min post-glucose administration. Similarly, insulin sensitivity was evaluated by administering insulin (0.8–1.2 units/kg body weight) to mice after a 6-h fasting period, with blood glucose measurements taken at the same time points before and after the insulin injection.

### Insulin sensitivity evaluation of liver, muscle, and adipose tissue

Tissue insulin responsiveness was assessed through the quantification of insulin-induced AKT phosphorylation in liver, skeletal muscle, and white adipose tissue. Following a 5-h fasting period, mice received an intraperitoneal (i.p.) injection of insulin (2 units/kg) or saline and were euthanized 10 min post-injection. Subsequent tissue collections from liver, muscle, and adipose sites were analyzed to determine the levels of phosphorylated AKT relative to total AKT.

### Histological analysis

Histological analysis involved hematoxylin and eosin (H&E) staining, where tissues were first encased in paraffin, then sliced into sections 5 μm thick. For the visualization of lipids, liver specimens were preserved in Tissue-Tek OCT compound (Leica) and cut into 15 μm sections with a Leica Cryostat for Oil Red O staining. Quantification of lipid droplet sizes was performed on H&E-stained slides from three distinct samples per group, utilizing ImageJ for diameter measurements.

### Quantification of liver TG in patients

Patients were scanned using a multidetector spiral CT (VCT, GE Healthcare) in a supine position, covering anatomical regions from the diaphragm to the pubic symphysis. Imaging parameters included a tube voltage of 120 kV, tube current of 240 mA, and a 5 mm slice thickness and interval. Further specifications involved a rotation time of 0.6 s, a helical pitch of 1.375, a field of view ranging from 35 to 40 cm, a 512 × 512 matrix, utilizing a standard reconstruction algorithm.

### Immunofluorescence microscopy

Cells (1 × 10^5^) were seeded on glass coverslips for 18 h, fixed in 4% paraformaldehyde, quenched with 0.1 M glycine, permeabilized in 0.2% BSA-0.05% saponin in PBS, and incubated with primary antibodies targeting Perilipin4, Bodipy (Sigma) followed by treatment with fluorescent-labeled secondary antibody and DAPI (Santa Cruz Biotechnology). Images were acquired on a Two-Photon Laser Confocal Microscope (Leica TCS SP8-MP).

### RNA isolation, reverse transcription PCR, and real-time PCR

RNA was extracted from tissue samples using TRIzol reagent (Takara Bio, Japan), and reverse transcription was achieved with PrimeScript *R. torques* Master Mix (Takara Bio, Japan). Quantitative real-time PCR analyses were conducted using SYBR Select Master Mix on an ABI 7300 sequence detector (Applied Biosystems, USA). The reverse transcription for *R. torques*-PCR utilized the TaqMan RNA Reverse Transcription Kit and 5x RNA primers, whereas qPCR employed the TaqMan Universal Master Mix II with 20x RNA primers in 10 μl volumes on a StepOnePlus Real-Time PCR System (ThermoFisher Scientific). Data normalization was performed against 18S rRNA levels in each sample, with U6 small nuclear RNA serving as the endogenous control for RNA normalization. The experimental replicates were processed in triplicate, and the 2^−ΔΔCt^ method was applied for data analysis.

### Subcellular fractionation

The evaluation of K-Ras's membrane anchoring involved separating cellular components using the Triton X-114 partition method (61). Initially, cell lysates were prepared. These lysates were then adjusted to a protein concentration of 1 mg/ml and mixed equally with 4% Triton X-114 for a 5-min incubation at 37 °C, aiming to solubilize and segregate the membrane's lipid-rich segments. Following this, the obtained subcellular fractions underwent immunoprecipitation using a K-Ras-specific antibody, followed by analysis through Western blotting.

### Protein extraction and western blot analysis

Cells or tissues underwent homogenization in RIPA buffer, enriched with both protease and phosphatase inhibitors (Roche Diagnostics). The protocol for western blotting adhered to standard practices, with protein concentrations determined *via* the BCA assay. Protein samples, ranging from 30 to 50 μg, were subjected to electrophoresis on a 10% SDS-PAGE, followed by transferal to PVDF membranes (Roche). Blocking was achieved using 5% non-fat milk in PBS for 1 hour at ambient temperature, before overnight incubation with specific primary antibodies at 4 °C. Secondary antibodies, at a 1:10,000 dilution in PBS, were applied for 1 hour at room temperature to detect the primary antibodies. Signal detection utilized an enhanced chemiluminescence method. Illustrations in the figures and supplementary figures represent findings from at least three separate experiments.

### Immunoprecipitation and analysis *via* Western blot

Following established protocols, immunoprecipitation targeted the K-Ras protein within cell lysates using a K-Ras-specific antibody. This complex was then captured using protein A/G plus agarose beads. Subsequently, proteins of interest were analyzed through Western blotting, specifically probing for K-Ras to determine its presence and quantity.

### Immunoprecipitation and western blot analysis

Immunoprecipitation was performed according to a standard protocol (62). K-Ras antibody was used to form an immune complex with K-Ras proteins in lysates and was immunoprecipitated down with protein A/G plus agarose. Finally, the equivalent protein samples were subjected to Western blot analysis against K-Ras.

### Chromatin Immunoprecipitation

Chromatin Immunoprecipitation (ChIP) using ZNF384 antibody involves initially treating cells with a cross-linking agent to stabilize protein-DNA interactions. Cells are then lysed to release chromatin. These fragments are incubated with ZNF384 antibodies to specifically bind the ZNF384 protein-DNA complexes. Magnetic beads coated with Protein A/G are used to capture the antibody-bound chromatin fragments. After several washes to remove non-specifically bound material, the protein-DNA complexes are eluted from the beads, and the cross-links are reversed to free the DNA. The DNA is then purified and analyzed by qPCR to identify the DNA-binding sites of ZNF384.

### Molecular docking

To analyze the binding affinities and modes of interaction between the small molucular and their targets, AutodockVina 1.2.2, a silico protein–ligand docking software, was employed (63). The molecular structures of ENMD-2076 were retrieved from PubChem Compound (https://pubchem.ncbi.nlm.nih.gov/). The 3D coordinates of KDR (PDB ID, 5EW3; resolution, 2.5 Å) and BIRC5(PDB ID, 4AOI; resolution, 1.9 Å) were downloaded from the PDB (http://www.rcsb.org/pdb/home/home.do). For docking analysis, all protein and molecular files were converted into PDBQT format with all water molecules excluded, and polar hydrogen atoms were added. The grid box was centered to cover the domain of each protein and to accommodate free molecular movement. The grid box was set to 30 Å × 30 Å × 30 Å, and the grid point distance was 0.05 nm. Molecular docking studies were performed by Autodock Vina 1.2.2 (http://autodock.scripps.edu/).

### Metabolite–protein interaction measurements

MST was performed with a Monolith NT.115 (*Nano Temper Co.*, München, Germany) according to the manufacturer’s instructions. Recombinant proteins were labeled with chemical dyes. A set of ten dilutions of MVL stock (1 mmol/L) was prepared with PBS, and the dilutions were mixed with GFP-ZNF384. After suctioning into capillary tubes, the fluorescence signals of the different proteins with different concentrations of MVL were detected and calculated.

### Quantification and statistical analysis

Blinding was implemented as deemed necessary and feasible to maintain objectivity during the study. Core personnel were blind to sample identities during data collection and analysis. No exclusion of samples or data occurred for statistical reasons. *In vitro* experiments were conducted in duplicates or triplicate to confirm the consistency of results. For animal studies, a minimum group size of six mice ensured a statistical power of at least 80%, with mice randomly allocated to various treatment groups. Statistical methodologies were outlined in the figure legends, where sample sizes were also specified. Statistical differences between two groups were determined using an unpaired two-tailed Student's *t* test after confirming data normality with Prism8 software (GraphPad software v8.0; Prism). A *p*-value ≤ 0.05 was considered significant, with the level of significance detailed in figure legends. For glucose and insulin tolerance tests, comparisons at each time point between groups were made using an unpaired two-tailed Student's *t* test.

## Data availability

All materials used in this study are either commercially available or obtained through collaboration and are available from the corresponding authors upon request. Further information and requests for resources and reagents should be directed to and will be fulfilled by the Lead Contact, Chao-jun Li (lichaojun@njmu.edu.cn).

## Supporting information

This article contains supporting information.

## Conflict of interest

The authors declare that they have no conflicts of interest with the contents of this article.
