# Supplementary material for: Elevated mevalonolactone from Ruminococcus torques contributes to metabolically unhealthy obesity development
Source: J Biol Chem. 2025 May 22;301(6):110281. doi: 10.1016/j.jbc.2025.110281 (PMC12214271; doi:10.1016/j.jbc.2025.110281)
Supplement: Supporting information [file mmc1.docx]

***Supporting information：Supplementary Figure***

**Elevated mevalonolactone from** ***Ruminococcus torques* contributes to Metabolically** **Unhealthy Obesity development**

**Running title: Elevated MVL from R. torques contributes to MUO**

Hong-Yu Nie^1^, Meng-Fei Zhao^1^, Tian-Yu Wu^3^, Ming-Jie Zou^1^, Yi-Ping Tang^1^, Xiao-Chen Wang^1^, Nan-Nan Wang^1^, Zi-Yue Zhou^1^, Yan Bi^1^, Yue Zhao^1^, Xi-Tai Sun^3^, Jing-Zi Zhang^1#^, Lei Fang^1#^, Chao-Jun Li^2#^

^1^ Ministry of Education Key Laboratory of Model Animal for Disease Study, Model Animal Research Center of the Medical School, Nanjing University, Nanjing, Jiangsu Province, China

^2^ State Key Laboratory of Reproductive Medicine and Offspring Heath, Center for Global Health, School of Public Health, Nanjing Medical University, Nanjing, China

^3^Department of Hepatobiliary Surgery, Affiliated Drum Tower Hospital, Medical School of Nanjing University, Nanjing, China.

#: Corresponding authors:

Dr. Jing-Zi Zhang (zhangjingzi@nju.edu.cn), Medical School of Nanjing University, Nanjing 210093, China.

Dr. Lei Fang ([njfanglei@nju.edu.cn](mailto:njfanglei@nju.edu.cn)), Medical School of Nanjing University, Nanjing 210093, China.

Dr. Chao-Jun Li ([lichaojun@njmu.edu.cn](mailto:lichaojun@njmu.edu.cn)), State Key Laboratory of Reproductive Medicine and Offspring Health of Nanjing Medical University, Nanjing 211166, China.

**
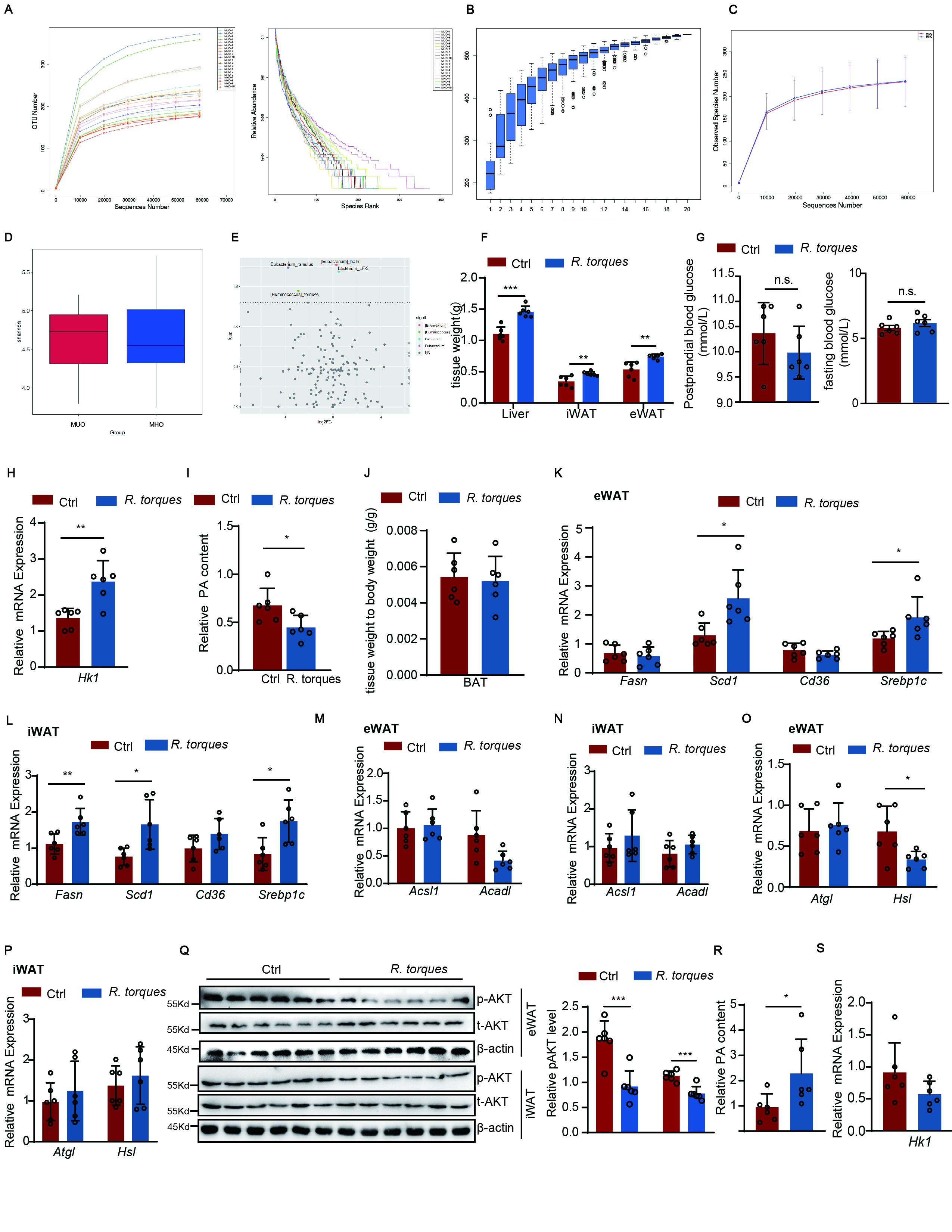
**

**Figure S1. Related to Figure 1. Increased *Ruminococcus torques* in MUO contributed to insulin resistance and Unhealthy lipid metabolisnm**

(A) Rarefaction Curve and Rank Abundance show that observed number of OTUs reached saturation in MUO and MHO patients.

(B) Species accumulation boxplot in MUO and MHO patients.

(C-D) Alpha diversity index analysis of differences between MHO and MUO groups.

(E) Alpha diversity indices analyzing MUO and MHO differential microbes.

(F) Actual weight of liver, iWAT and eWAT in mice with Ctrl and *R. torques*-treated (n = 6 mice per group).

(G) Postprandial blood glucose and Fasting blood glucose in mice with Ctrl and *R. torques*-treated (n = 6 mice per group).

(H-I) Liver pyruvic acid (PA) levels (H) and expression of *Hk1* (I) in Ctrl and *R. torques*-treated mice (n = 6 mice per group).

(J) Percentage of the BAT weight relative to the whole-body weight of *R.torques* treated mice (n = 6).

(K-L)Expression of genes related to lipogenesis and FFA uptake in eWAT (K) and iWAT (L) of mice treated with Ctrl and *R. torques* (n = 6).

(M-N) Expression of genes related to fatty acid oxidation in in eWAT (M) and iWAT (N) of mice treated with Ctrl and *R. torques* (n = 6 mice per group).

(O-P) Expression of genes related to lipolysis in eWAT (O) and iWAT (P) of mice treated with Ctrl and *R. torques* (n = 6 mice per group).

(Q) Insulin-stimulated phosphorylation of AKT in eWAT and iWAT of mice treated with *R. torques*, pAKT bands were analyzed by densitometry and results were normalized to AKT.

(R-S) eWAT pyruvic acid (PA) levels (R) and expression of *Hk1*(S) in Ctrl and *R. torques*-treated mice (n = 6 mice per group).

All experiments were repeated at least twice with similar results. *p < 0.05, **p < 0.01, ***p < 0.001; *n.s.*, no significant difference. Data are represented as mean ± SD. Two-sided Student’s t-test.


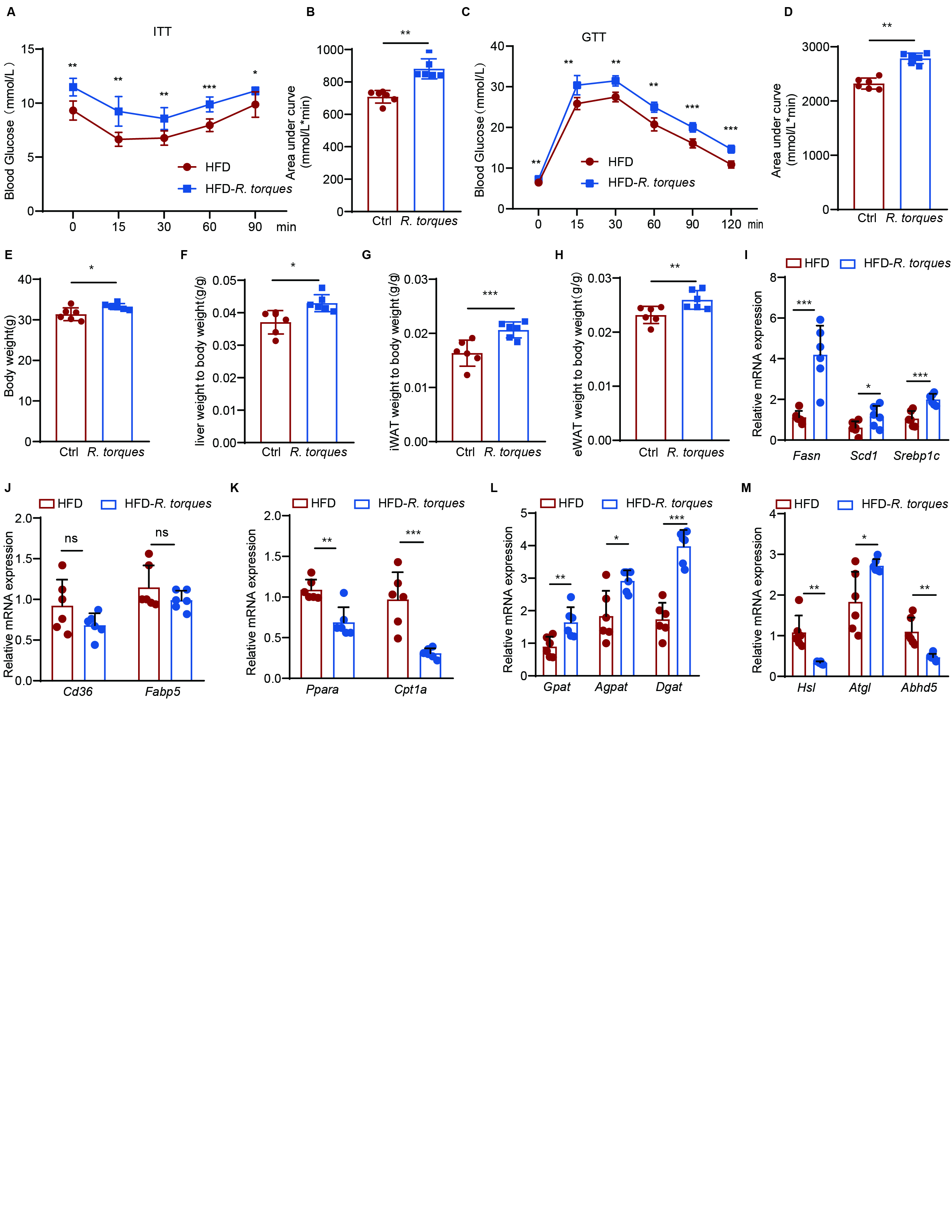


**Figure S2. Related to Figure 1. Increased *Ruminococcus torques* in MUO contributed to insulin resistance and Unhealthy lipid metabolisnm under HFD-feeding.**

(A-D) ITT (A-B) and GTT (C-D) in Ctrl and *R. torques*-treated mice with HFD (n = 6 mice per group).

(E) Body weight of Ctrl and *R. torques*-treated mice with HFD-fed (n=6).

(F) Liver weight to body weight ratio in Ctrl and *R. torques*-treated mice fed with HFD (n = 6 mice per group).

(G-H) iWAT (G) and eWAT (H) weight to body weight ratio in Ctrl and *R. torques*-treated mice under HFD feeding (n = 6 mice per group).

(I-M) Expression of genes related to lipogenesis (I), fatty acid uptake (J), fatty acid oxidation (K), TG synthesis (L) and lipolysis (M) in the liver of HFD-mice treated with PBS and *R. torques* (n = 6).

All experiments were repeated at least twice with similar results. *p < 0.05, **p < 0.01, ***p < 0.001; *n.s.*, no significant difference. Data are represented as mean ± SD. Two-sided Student’s t-test.

**
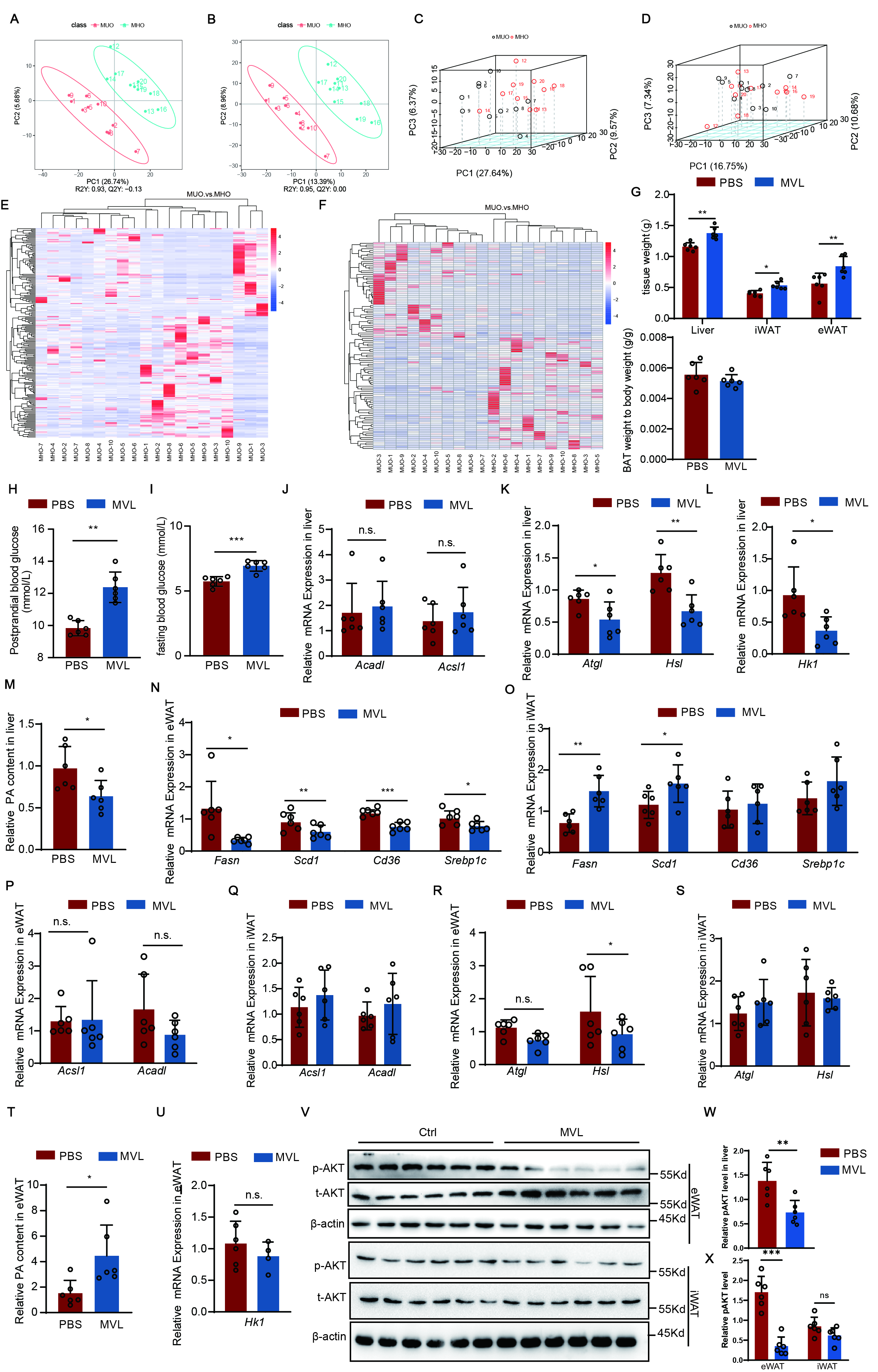
Figure S3. Related to Figure 2. Elevated levels of mevalonate lactone (MVL), induced by Ruminococcus torques, were associated with disrupted glucose and lipid metabolism.**

(A-B) PC1 vs. PC2 biplot of the negative iron mode (A) and the positive iron mode (B) metabolites based on features from the final PLS-DA model.

(C-D) Three-dimensional PCoA analysis of MUO and MHO Negative (C) and positive (D) fecal metabolism groups.

(E-F) Heatmap plot of the negative mode (E) and the positive iron mode (F) metabolites in patients with MUO and MHO.

(G) Actual weight of liver, iWAT, eWAT in mice with Ctrl or *R. torques*-treated. Percentage of the BAT weight relative to the whole-body weight of MVL-treated mice (n = 6).

(H-I) Postprandial blood glucose (H) and Fasting blood glucose (I) in Ctrl or MVL-treated (n = 6 mice per group).

(J-K) Expression of genes related to fatty acid oxidation (J) and lipolysis (K) in liver of mice treated with MVL (n = 6 mice per group).

(L) The relative mRNA expressions of *Hk1* in the hepatocyte from the indicated groups (n=6).

(M) Liver pyruvic acid (PA) levels in Ctrl and MVL-treated mice (n = 6 mice per group).

(N-O) Expression of genes related to lipogenesis and FFA uptake in eWAT (N) and iWAT (O) of mice treated with PBS and MVL (n = 6).

(P-Q) Expression of genes related to fatty acid oxidation in eWAT (P) and iWAT (Q) of mice treated with PBS and *R. torques* (n = 6 mice per group).

(R-S) Expression of genes related to lipolysis in eWAT (R) and iWAT (S) of mice treated with PBS and *R. torques* (n = 6 mice per group).

(T) eWAT pyruvic acid (PA) levels in Ctrl and *R. torques*-treated mice (n = 6 mice per group).

(U) Expression of *Hk1* in eWAT of mice treated with Ctrl and MVL (n = 6 mice per group).

(V) Insulin-stimulated phosphorylation of AKT in the hepatocytes treated with MVL.

(W-X) pAKT bands in *Figure2T* (W), *Figure S3V* (X)were analyzed by densitometry and results were normalized to AKT.

All experiments were repeated at least twice with similar results. *p < 0.05, **p < 0.01, ***p < 0.001; *n.s.*, no significant difference. Data are represented as mean ± SD. Two-sided Student’s t-test or two-way ANOVA was used.


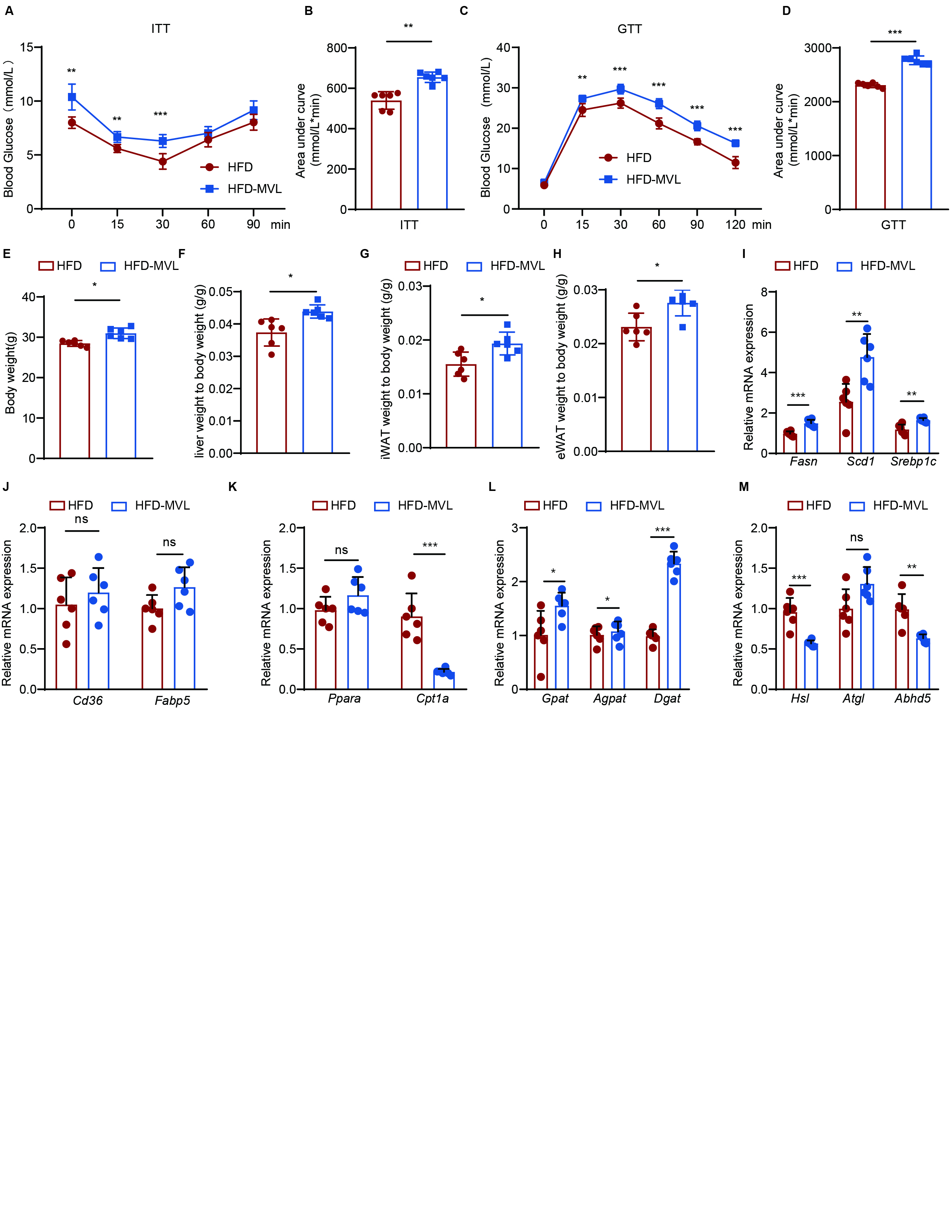


**Figure S4. Related to Figure 2. Elevated levels of mevalonate lactone (MVL), induced by Ruminococcus torques, were associated with disrupted glucose and lipid metabolism under HFD-feeding.**

(A-D) ITT (A-B) and GTT (C-D) in PBS and MVL-treated mice with HFD (n = 6 mice per group).

(E) Body weight of PBS and MVL-treated mice with HFD-fed (n = 6 mice per group).

(F) Liver weight to body weight ratio in PBS and MVL-treated mice fed with HFD (n = 6 mice per group).

(G-H) iWAT (G) and eWAT (H) weight to body weight ratio in PBS and MVL-treated mice under HFD feeding (n = 6 mice per group).

(I-M) Expression of genes related to lipogenesis (I), fatty acid uptake (J), fatty acid oxidation (K), TG synthesis (L) and lipolysis (M) in the liver of HFD-mice treated with PBS and MVL (n = 6).

All experiments were repeated at least twice with similar results. *p < 0.05, **p < 0.01, ***p < 0.001; n.s., no significant difference. Data are represented as mean ± SD. Two-sided Student’s t-test.

**
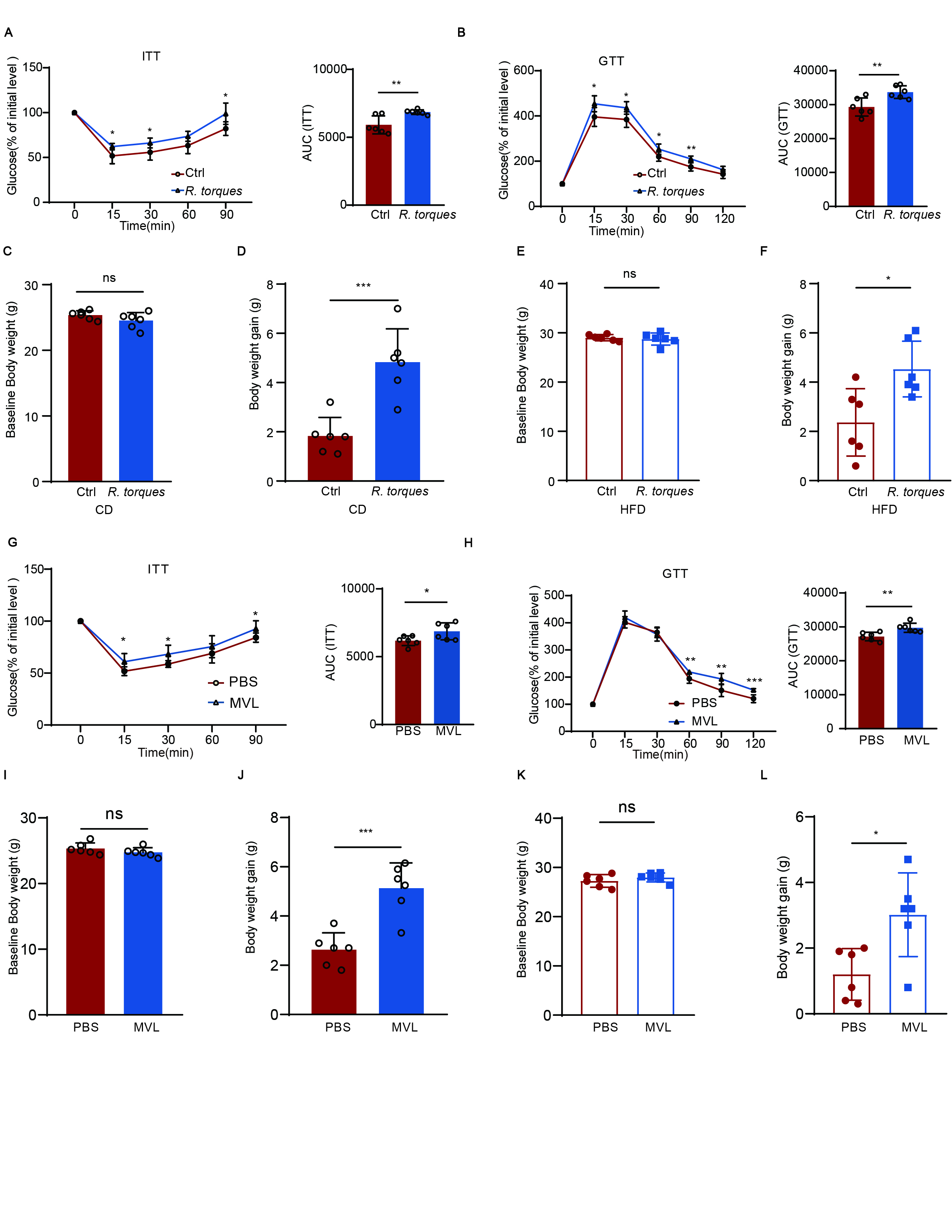
**

**Figure S5. Effects of *Ruminococcus torques* and MVL treatment on body weight gain and insulin resistance in mice under chow diet (CD) and high-fat diet (HFD) conditions.**

(A-B) Insulin tolerance test (ITT, A) and glucose tolerance test (GTT, B) in control and *R. torques*-treated mice (n = 6 per group).

(C) No significant difference in baseline body weight between groups prior to *R. torques* administration (n = 6 per group).

(D) *R. torques* treatment significantly increased body weight gain compared to controls under CD feeding (n = 6 per group).

(E) Under HFD conditions, no significant difference in baseline body weight was observed between CTRL and *R. torques*-treated groups prior to treatment (n = 6 per group).

(F) *R. torques*-treated mice exhibited significantly greater body weight gain than controls under HFD feeding (n = 6 per group).

(G-H) Insulin tolerance test (ITT, G) and glucose tolerance test (GTT, H) in PBS and MVL-treated mice (n = 6 per group).

(I) No significant difference in baseline body weight between groups prior to MVL administration (n = 6 per group).

(J) MVL treatment significantly increased body weight gain compared to controls under CD feeding (n = 6 per group).

(K) Under HFD conditions, no significant difference in baseline body weight was observed between PBS and MVL-treated groups prior to treatment (n = 6 per group).

(L) MVL-treated mice exhibited significantly greater body weight gain than controls under HFD feeding (n = 6 per group).

All experiments were repeated at least twice with similar results. *p < 0.05, **p < 0.01, ***p < 0.001; n.s., no significant difference. Data are represented as mean ± SD. Two-sided Student’s t-test was used.

**
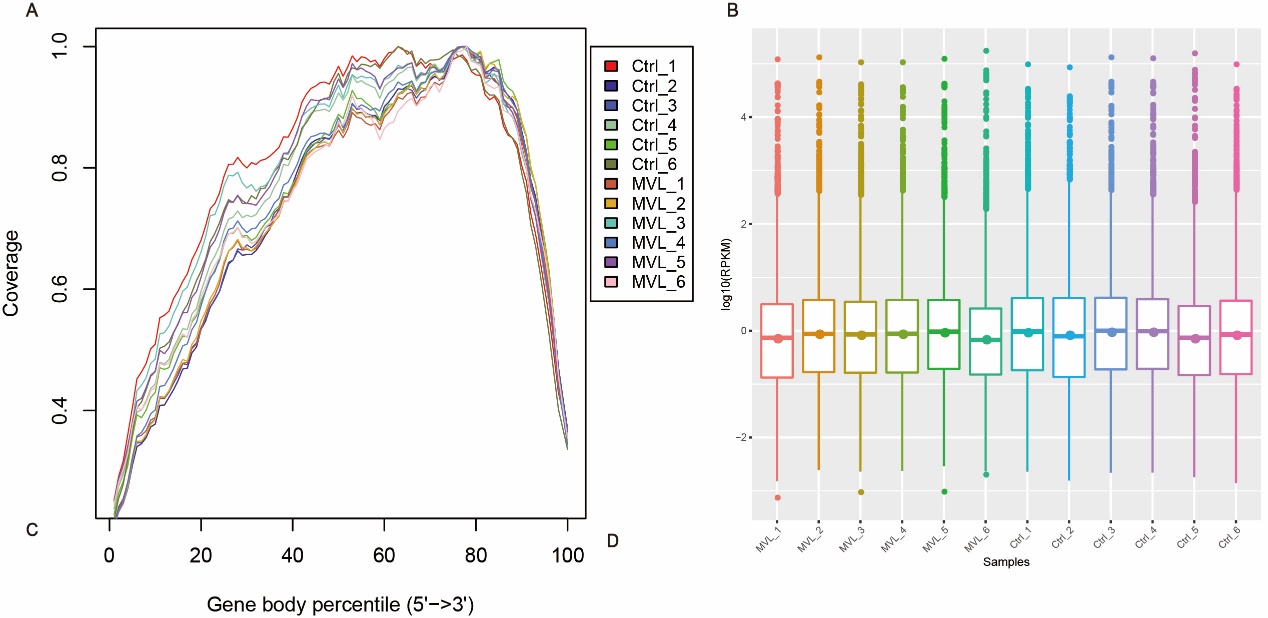
**

**Figure S6. Related to Figure 3. MVL alters the metabolic pattern of the liver**

(A) Reads coverages of gene body percentile of sequenced samples. Ctrl: mice treated with PBS, MVL: mice treated with MVL (n=6).

(B) Boxplot of RPKM normalized expression for 8 samples from the liver of mice treated with MVL. Samples with the same color were the same RNA extract but prepared by either the PolyA or NuGEN protocol.

**
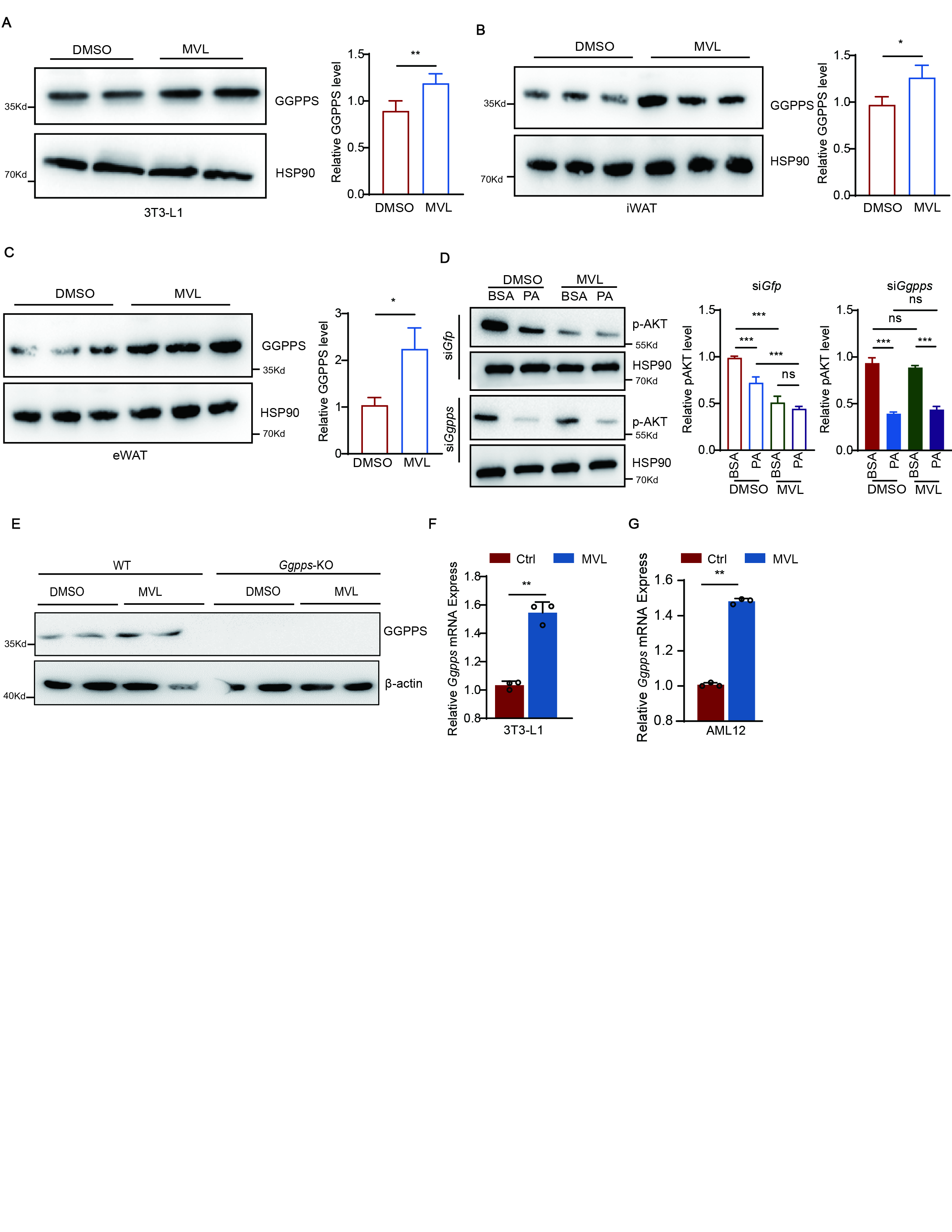
**

**Figure S7. Related to Figure 4. MVL treatment induced insulin resistance and increased cholesterol synthesis in the mice**

(A) The protein level of GGPPS in MVL-treated cells (3T3-L1). GGPPS bands were normalized to HSP90.

(B-C) The protein level of GGPPS in iWAT (B) and eWAT (C) of MVL-treated mice. GGPPS bands were normalized to HSP90 (n=3).

(D) Western blotting was used to detect p-AKT in AML12 cells with si*Ggpps* and MVL treatment. P-AKT bands were normalized to HSP90.

(E) Western blotting was used to detect GGPPS in *Ggpps*-KO mice.

(F-G) Expression of *Ggpps* in MVL treated cells (3T3-L1(D) and AML12 (E)). (n=3)

All experiments were repeated at least twice with similar results. *P < 0.05, **P < 0.01, ***P < 0.001, *n.s.*, no significant difference. Data are represented as mean ± SD. two-way ANOVA.


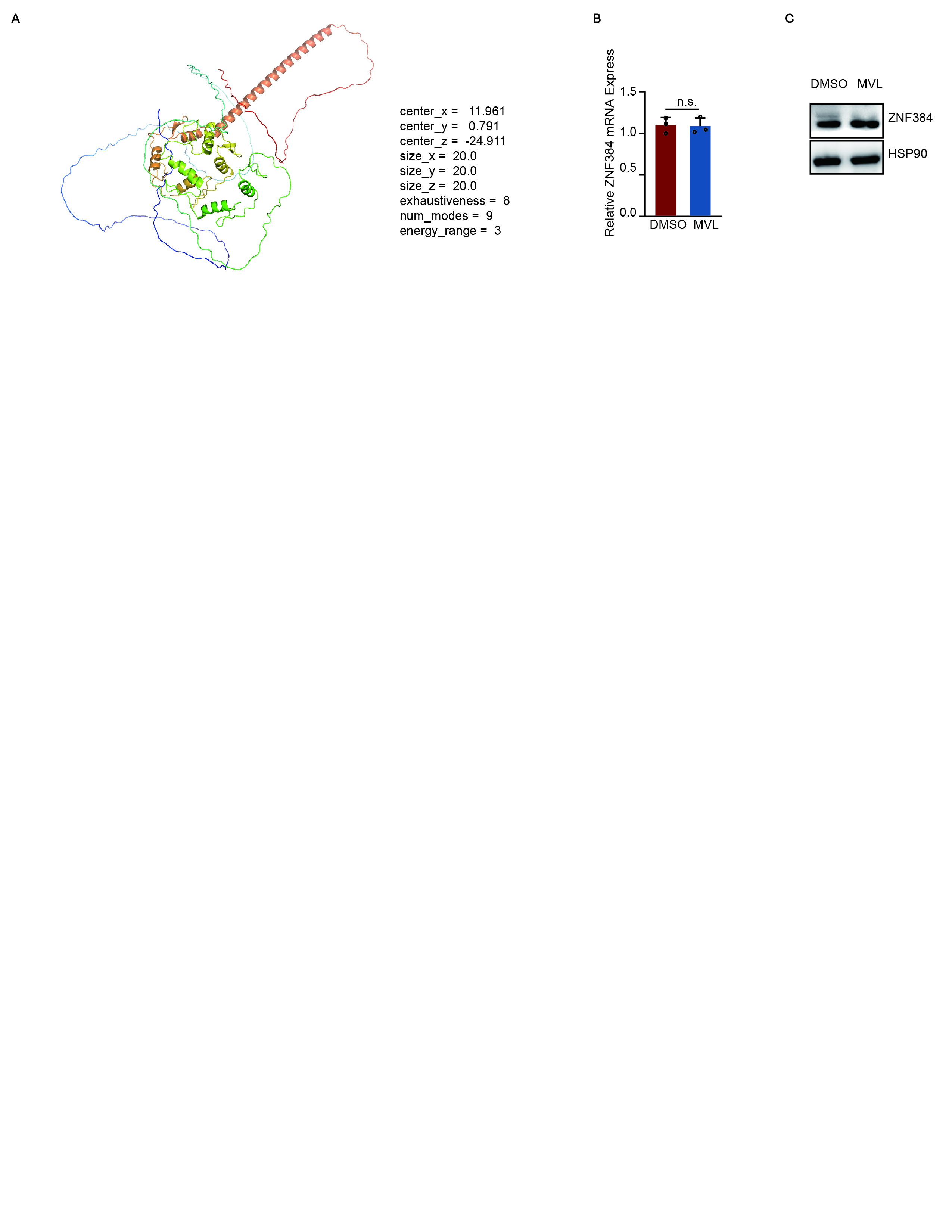


**Figure S8. Related to Figure 6.** **MVL binding to ZNF384 affects its nuclear localization**

(A) Download protein structures from the UniProt database, subsequently visualize them separately using PyMOL, and then process them using Mgtools 1.5.6 to remove water molecules, add hydrogen atoms, calculate charges, and merge nonpolar hydrogens, among other treatments.

(B) The effect of MVL treatment on Znf384 RNA levels was assessed.

(C) The impact of MVL treatment on Znf384 protein levels was evaluated.

All experiments were repeated at least twice with similar results. *p < 0.05, **p < 0.01, ***p < 0.001; *n.s.*, no significant difference. Data are represented as mean ± SEM. Two-sided Student’s t-test.
